# Identifying large-scale recombination and capsular switching events in *Streptococcus agalactiae* strains causing disease in adults in the UK between 2014 and 2015

**DOI:** 10.1099/mgen.0.000783

**Published:** 2022-03-15

**Authors:** Uzma Basit Khan, Elita Jauneikaite, Robert Andrews, Victoria J. Chalker, Owen B. Spiller

**Affiliations:** ^1^​ Division of Infection and Immunity, School of Medicine, Cardiff University, Cardiff, UK; ^2^​ Department of Infectious Disease Epidemiology, School of Public Health, Imperial College London, London, UK; ^3^​ NIHR Health Protection Research Unit in Healthcare Associated Infections and Antimicrobial Resistance, Department of Infectious Disease, Faculty of Medicine, Imperial College London, London, UK; ^4^​ Systems Immunity University Research Institute, Division of Infection and Immunity, School of Medicine, Cardiff University, Cardiff, UK; ^5^​ National Infection Service, United Kingdom Health Security Agency, Colindale, London, UK; ^‡^​ Present address: Parasites and Microbes, Wellcome Sanger Institute, Wellcome Genome Campus, Hinxton CB10 1SA, UK

**Keywords:** capsular serotype, epidemiology, group B *Streptococcus*, MLST, whole-genome sequencing

## Abstract

Cases of invasive group B streptococcal infection in the adult UK population have steadily increased over recent years, with the most common serotypes being V, III and Ia, but less is known of the genetic background of these strains. We have carried out in-depth analysis of the whole-genome sequences of 193 clinically important group B *Streptococcus* (GBS) isolates (184 were from invasive infection, 8 were from non-invasive infection and 1 had no information on isolation site) isolated from adults and submitted to the National Reference Laboratory at the UK Health Security Agency between January 2014 and December 2015. We have determined that capsular serotypes III (26.9%), Ia (26.4%) and V (15.0%) were most commonly identified, with slight differences in gender and age distribution. Most isolates (*n*=182) grouped to five clonal complexes (CCs), CC1, CC8/CC10, CC17, CC19 and CC23, with common associations between specific serotypes and virulence genes. Additionally, we have identified large recombination events mediating potential capsular switching events between sequence type (ST)1 serotype V and serotypes Ib (*n*=2 isolates), II (*n*=2 isolates) and VI (*n*=2 isolates); between ST19 serotype III and serotype V (*n*=5 isolates); and between CC17 serotype III and serotype IV (*n*=1 isolate). The high genetic diversity of disease-causing isolates and multiple recombination events reported in this study highlight the need for routine surveillance of the circulating disease-causing GBS strains. This information is crucial to better understand the global spread of GBS serotypes and genotypes, and will form the baseline information for any future GBS vaccine research in the UK and worldwide.

## Impact Statement

This study is, to the best of our knowledge, the first study to report on in-depth genomic analysis of the disease-causing group B *Streptococcus* (GBS) in the adult population in the UK. We describe the most common serotype–genotype combinations, including multilocus sequence types and major virulence gene combinations for the specific serotypes, found in our dataset. Importantly, we report on various potential capsular type switching events caused by recombination for certain GBS genotypes.

## Data Summary

The raw sequencing reads from the 193 group B *Streptococcus* (GBS) isolates used in this study are available from the European Nucleotide Archive under the project accession number PRJEB18093, and genome assemblies are available under project accession number PRJEB48955. Accession numbers for individual GBS isolates are provided in Table S1 (available with the online version of this article).

## Introduction

*Streptococcus agalactiae* (or group B *Streptococcus *, GBS) is a Gram-positive encapsulated commensal pathogen of the human genitourinary and gastrointestinal tract, which can cause disease under certain circumstances (e.g. reduced immune function). GBS is well established as a leading cause of bacterial sepsis and meningitis in neonates [1], and is increasingly associated with invasive infections in adults [2–4]. In the UK, cases of invasive GBS (iGBS) in the adult population, particularly the elderly and women of childbearing age, have increased over the last 15 years; with previous studies reporting annual incidences from 0.92 to 2.39 per 100 000 population during 1991–2010 [3], increasing to 3.48 per 100 000 population during 2015–2016 [5].

GBS is sub-divided into ten serotypes (Ia, Ib, II–IX) based on capsular polysaccharides. However, only five serotypes, Ia, Ib, II, III and V, account for 93–99% of cases of neonatal and adult infections globally [6]; with serotypes V and Ia accounting for 58% of elderly iGBS, and serotype III dominating in neonatal iGBS cases [6]. Currently, there are three main GBS vaccines in development: two multi-capsular polysaccharide vaccines, trivalent, covering serotypes Ia, Ib and III [7] and hexavalent, covering serotypes Ia, Ib, II, III, IV and V [8, 9]; and a multivalent adjuvanted protein vaccine (NCT03807245). With these GBS vaccines on the horizon, questions arise whether implementation of GBS polysaccharide-based vaccines will drive serotype replacement, or capsular switching, which has been reported for other vaccine-preventable bacterial pathogens, such as *Streptococcus pneumoniae* [10, 11]. Increasing numbers of GBS genomic epidemiology studies allow us to get an insight into capsular switching events. To date, a higher frequency of capsular switching events has been recorded in the sequence type (ST)1 GBS lineage [12, 13], which is dominated by serotype V and commonly found in non-pregnant adult iGBS cases, and recombination of capsular sites for serotypes Ib, II and IV have been reported for this lineage [14, 15]. On the contrary, the ST17 lineage is uniformly dominated by serotype III and recombination events that have been reported include members of clonal complex (CC)17 [16] rather than the ST17 lineage. As GBS has the potential to achieve capsular switching, there is possibility for the less common serotypes to become established in iGBS. Therefore, structured longitudinal surveillance of carriage and clinical GBS strains is important to help underpin the prevalence and conditions required for capsular switching events in the GBS population, and the implications of such events on clinical disease incidence and the GBS vaccine implementation policy. In the UK, recently, a detailed genomic surveillance of GBS isolates causing early onset and late-onset GBS disease in infants has been carried out that uncovered clusters of infection [17]; however, detailed information on genomic features of adult GBS isolates in the UK is lacking. Here, we used whole-genome sequencing to characterize disease-causing GBS strains from the adult population in the UK and report putative capsular switching events that occurred in this GBS population.

## Methods

### Genomic sequences of clinical GBS isolates

Sterile site GBS isolates from adults are, where possible, routinely submitted to the UK Health Security Agency (UKHSA) reference laboratory as part of national surveillance. In 2014 to 2015, isolates were mainly submitted from laboratories in England, with a small proportion of submissions from elsewhere in the UK. Isolates received are from patients of all ages, with the majority from neonates (estimated >90%) and women of child-bearing age, with an estimated total adult isolate submission proportion of less than 10% of all invasive cases. All available GBS isolates from invasive and non-invasive adult disease cases submitted to UKHSA between January 2014 and December 2015 were whole-genome sequenced and used in this study. Available anonymised clinical data included information on age range and sex of the patient, year, and sample type where GBS was isolated. DNA was extracted using a Wizard genomic DNA purification kit (Promega). A Nextera XT DNA library preparation kit (Illumina) was used to prepare multiplexed DNA sequencing libraries. Whole-genome sequencing was undertaken using the Illumina HiSeq 2500 system (Illumina) and 2×100 bp paired-end mode.

### Assembly and annotation

High-quality reads were *de novo* assembled using Spades v3.9.0 [18]. Pilon v1.22 [19] and flash v1.2.11 [20] were used to improve draft genome assemblies by correcting bases, fixing mis-assemblies and gap filling. quast v2.1 [21] was used to summarize statistics for genome assemblies (Table S1). Genome annotation was performed using Prokka v1.12 [22] and Geneious v11.0.5 (Biomatters) [23].

### Capsular serotyping and multilocus sequence typing (MLST)

GBS capsular serotype was assigned *in silico* as described previously [24]. Further validation of capsular serotype was performed using the blast tool in Geneious v11.0.5 (Biomatters) and sequence-based serotype allocation using serotype references as follows: serotype Ia (AB028896.2) [25], serotype Ib (AB050723.1) [26], serotype II (LT671985.1) [24], serotype III (AF163833.1) [27], serotype IV (AF355776.1) [28], serotype V (AF349539.1) [28], serotype VI (AF337958.1) [28], serotype IX (LT671992.1) [24]. MLST ST was assigned using srst2 v0.2.0 [29]. Alleles and STs not previously described were deposited in the *S. agalactiae* MLST database (https://pubmlst.org/sagalactiae/) [30]. PHYLOViZ v2.0 [31] and the goeBURST algorithm were used to establish relationships between STs using ‘3’ as the minimum single locus variant (SLV) count for subgroup definition and ‘6’ as the minimum number of identical loci for group definition.

### Genes for pilus island, *hvgA* and surface proteins

The presence of surface protein genes, *alp*2 and *alp*3, the *hvgA* gene, and the pilus island *PI-1*, *PI-2a* and *PI-2b* genes were determined *in silico* using oligonucleotide primers published elsewhere [32, 33]. Presence and absence of virulence genes alpha and alpha-like surface protein (*bca*, *cba*, *rib*) and bacterial adhesin, *bib*A, was evaluated using srst2 v0.2.0 [29] and Geneious 11.0.5 [23].

### Phylogenetic analysis

Raw reads were mapped to a reference genome and SNPs were called using snippy v3.2 (https://github.com/tseemann/snippy). For the SNP-based genomic analysis, we have selected the genetically closest closed reference genome available from the National Center for Biotechnology Information (NCBI) (available from: https://www.ncbi.nlm.nih.gov/genome/browse/#!/prokaryotes/186/) that matched our isolates by the most common MLST genotype and serotype observed within the CCs as follows: for the analysis of CC1 isolates, reference sequence SS1 (NZ_CP010867.1) was used; for the CC17 reference sequence COH1 (NZ_HG939456.1); for the CC19 reference sequence SG-M25 (CP021867); for the CC8 reference sequence Sag37 (NZ_CP019978.1); and for the CC23 reference sequence FDAARGO_512 (NZ_CP033822.1). Gubbins v.2.3.4 [34] was used to identify and remove recombination regions prior to reconstructing a maximum-likelihood phylogenetic tree using core SNP alignment and RAxML [35]. Phylogenetic trees were visualized using the Interactive Tree of Life (iTOL) (https://itol.embl.de/) [36].

### Recombination analysis to detect potential capsular switching events

Gubbins [34] was used to detect recombination hotspots and the blast ring image generator (brig) [37] was used to visualize these recombination events. Recombination analysis was performed against a reference genome: in non-serotype V ST1 isolates (*n*=6) using reference sequence SS1 (NZ_CP010867.1), for CC17 serotype IV *hvgA* positive isolate (*n*=1) reference sequence COH1 (NZ_HG939456.1) was used, for serotype V ST19 isolates (*n*=5) reference sequence SG-M25 (CP021867) was used, and for serotype V ST498 isolates (*n*=2) reference sequence FDAARGO_512 (NZ_CP033822.1) was used.

### Statistical analysis

Chi-square and Fisher’s exact tests were used to assess associations between GBS serotype and patient gender. Data were visualized using ggplot2 [38] and R version 4.0.2 [39].

## Results

### Population characteristics and serotype distribution

**Fig. 1.**
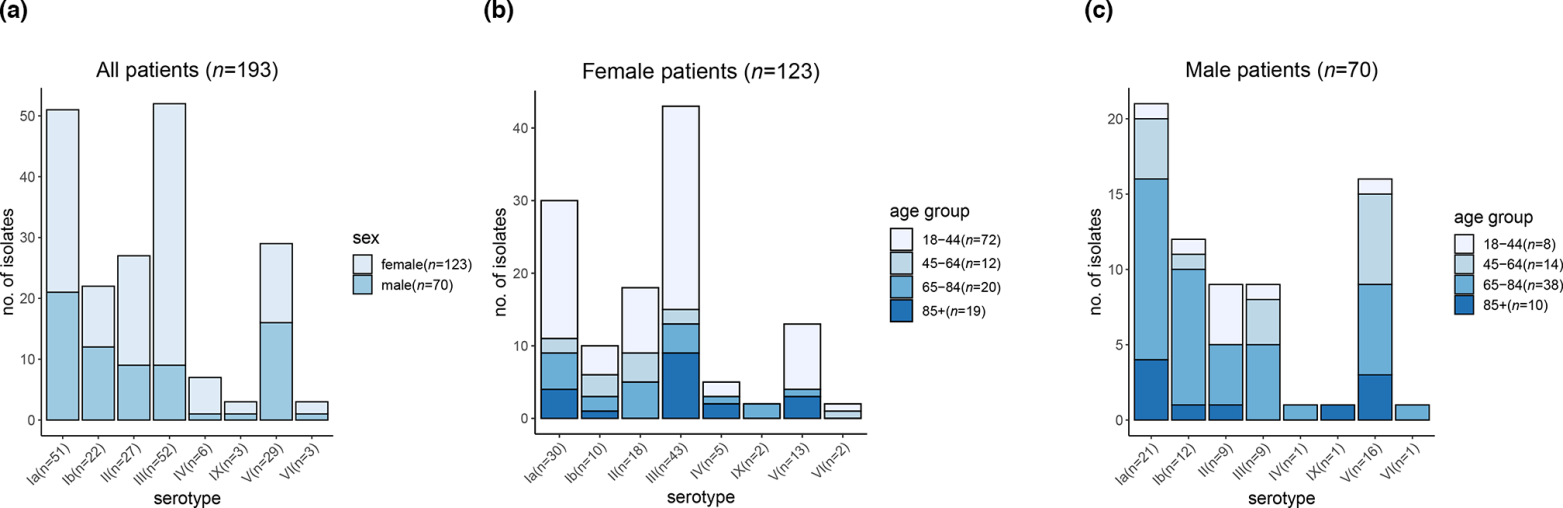
Summary of demographic information available for 193 *S. agalactiae* strains submitted to the UKHSA between January 2014 and December 2015. (**a**) Overall number of GBS serotypes observed in the dataset segregated by sex; (**b**) graphical distribution of GBS serotypes identified in female patients, segregated by age group; (**c**) graphical distribution of GBS serotypes identified in male patients, segregated by age group.

In total, 193 GBS isolates, 184 from iGBS disease cases (isolates from blood, *n*=178; cerebrospinal fluid, *n*=1; aortic valve, *n*=1; amniotic membrane, *n*=1; placenta, *n*=3), and 8 from non-invasive cases (isolates from pus, *n*=2; throat swab, *n*=1; tissue, *n*=2; vaginal swab, *n*=1; wound swab, *n*=1; abscess, *n*=1) and 1 isolate had no information on isolation site, were available for analysis (Table S2). In this dataset, age of the patients ranged from 20 to 100 years (median age 59 years) with 63.7% (*n*=123/193) of GBS isolates from female adult patients. As expected, due to the potential for post-partum infection in females, a lower median age group was observed (median age 38 years old, interquartile range 41, 31.5–72.5) (Fig. 1a) compared to males (median age 72.5 years old, interquartile range 19.75, 61.25–81). In total, 58.5% (*n*=72/123) of GBS strains from female patients were isolated from younger women aged 18 to 44 years old, relative to women over 44 years of age (41.5%, *n*=51/123) (Fig. 1b), whereas only 11.4% (*n*=8/70) were isolated from male patients 18–44 years old (Fig. 1c).

In total, eight serotypes were identified: III (26.9%, *n*=52/193), Ia (26.4%, *n*=51/193), V (15%, *n*=29/193), II (14.0%, *n*=27/193), Ib (11.4%, *n*=22/193), IV (3.1%, *n*=6/193), VI (1.6%, *n*=3/193) and IX (1.6%, *n*=3/193). We have observed that serotype III was the dominant serotype in female patients with 43 isolates reported from this group compared to 9 isolates reported in the male patient group (Fig. 1b, c), this suggested that serotype III is more likely to be associated with female patients [X^2^(1, *n*=193) =11.07; *P*=0.0009]. Serotype Ia was the second most common serotype identified in female patients (*n*=30/123; Fig. 1b) and it was the top serotype identified in male patients (*n*=21/70; Fig. 1c). Serotype V was the second most common serotype in male patients (*n*=16/70; Fig. 1c), while in female patients serotype V ranked as the fourth most common serotype (*n*=13/123; Fig. 1b), this suggested that serotype V was potentially more commonly causing infections in male patients [X^2^(1, *n*=193)=5.3; *P*=0.02]. Serotypes III and Ia were more common among female patients, especially in the younger age group (18–44 years old), with the exception for the 45–64 years age group, where serotypes II and Ib pre-dominated, though this group contains a small number of isolates in comparison to other age groups (Fig. 1b). To confirm these observed serotype and gender or age group associations, further analysis on a larger longitudinal adult GBS dataset is needed.

### Genotypes and virulence genes

Of the 47 STs found, 5 were predominant: ST23 (*n*=40/193), ST8 (*n*=29/193), ST17 (*n*=29/193), ST1 (*n*=23/193) and ST19 (*n*=20/193). Additionally, 15 novel STs were identified (Table S2). The STs were further grouped into five main CCs: CC23 (27.5%, *n*=53/193), CC1 (18.1%, *n*=35/193), CC8/CC10 (15.0%, *n*=29/193), CC17 (16.6%, *n*=32/193) and CC19 (17.1%, *n*=33/193) (Table S2). The majority of CC23 strains were serotype Ia (90.6%, *n*=48/53), while CC1 isolates were mainly serotype V (57.1%, *n*=20/35) and predominantly ST1/*alp*3-positive (*n*=17/20). Serotypes Ib and II were mainly grouped within CC8/CC10, with a majority of the isolates being ST8 (*n*=9/29) and ST12 (*n*=13/29) (Table S2). Most serotype III isolates aligned either with CC19 (*n*=17/33) isolates or hypervirulent clone CC17 (*n*=31/32) isolates, all of which, bar one isolate of ST1220 serotype Ib, possessed the *hvgA* gene (Table S2). All 193 GBS isolates harboured at least one pilus island, *PI-1+PI-2a* was the most frequent combination observed (81.9%, *n*=158/193), with only CC17 being dominated by the presence of the *PI-1+PI-2b* gene (96.9%, *n*=31/32) combination. The *rib* gene was found mainly in CC17 (96.9%, *n*=31/32) and CC19 (81.8%, *n*=27/33) isolates (Table S2).

### Phylogenetic relationship between GBS strains

**Fig. 2.**
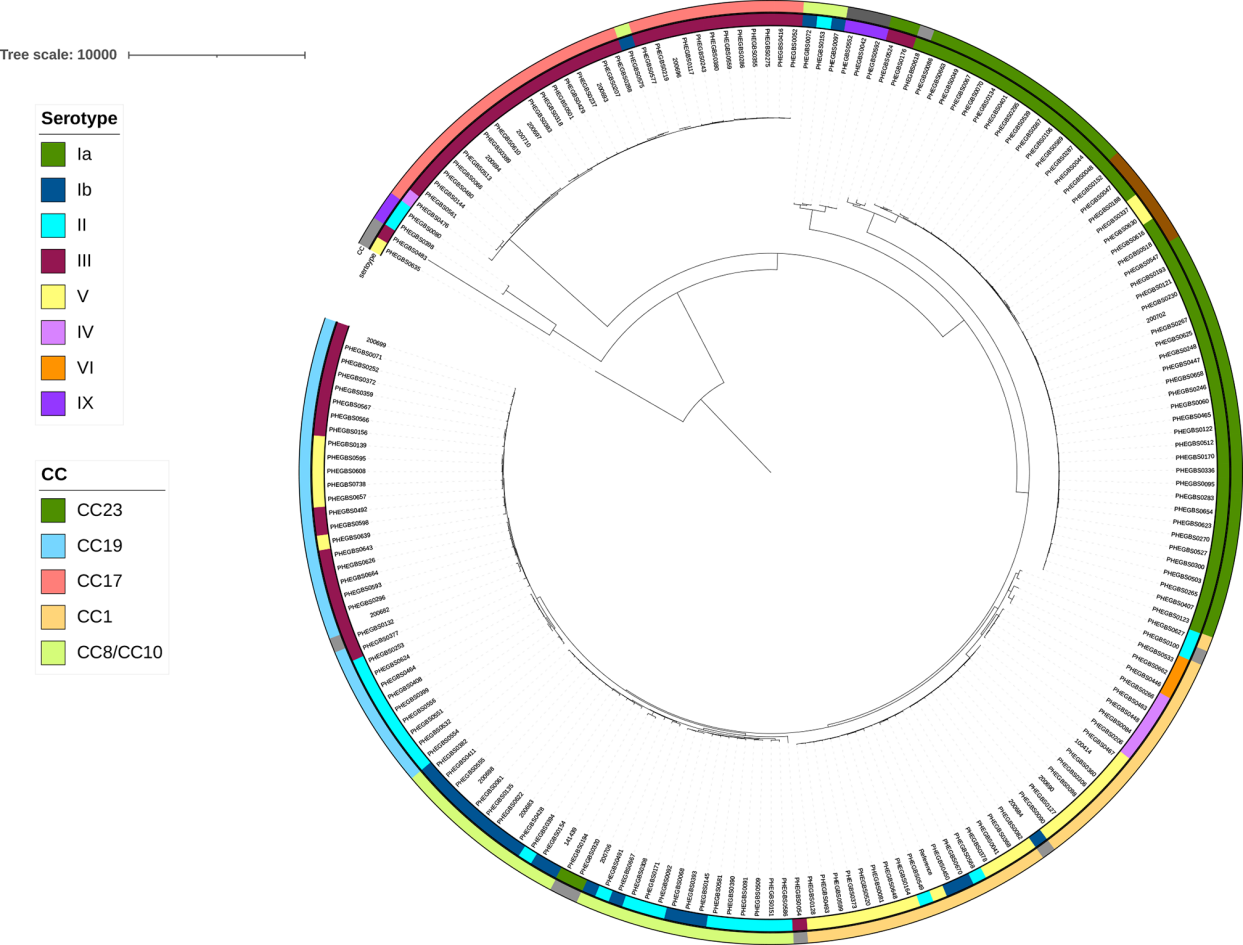
Phylogenetic relationship between GBS isolates causing disease in the adult population in the UK. A total of 193 genomes was analysed for the study. Mid-point rooted maximum-likelihood phylogeny tree built using SNPs after correcting for recombination. The inner ring indicates one of the eight serotypes identified amongst 193 GBS isolates in this study. The outer ring indicates five major CCs, CC23, CC1, CC19, CC17 and CC8/CC10, identified in the study; purple indicates CC22/CC1213, brown indicates CC24/CC498, dark grey indicates CC130/CC1216, and light grey colour indicates isolates identified as singletons based on *goe*BURST SLV analysis. The scale bar represents a distance of 10000 point mutations.

**Fig. 3.**
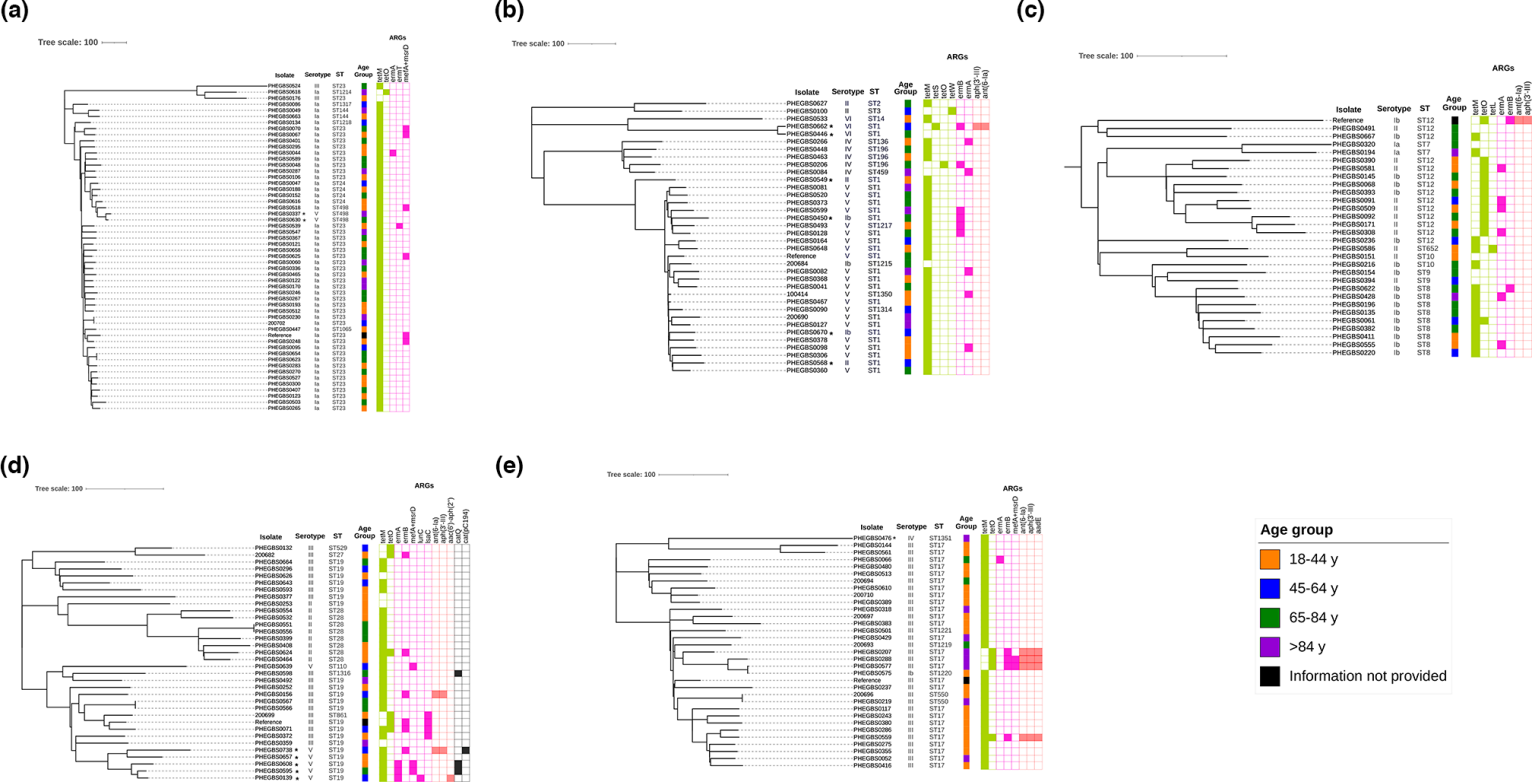
Phylogenetic relationship between GBS isolates within major CCs causing disease in the adult population in the UK. Phylogenetic analysis indicates strains belong to the five major CCs identified in this study. (**a**) Phylogenetic relationship between GBS isolates belonging to CC23. Fifty-three isolates grouped to CC23 and are represented by a mid-point rooted maximum-likelihood phylogeny tree using 1570 core SNPs called from reference sequence FDAARGOS 512 (NZ_CP033822.1). (**b**) Phylogenetic relationship between GBS isolates belonging to CC1. Thirty-five isolates grouped to CC1 and are represented by a mid-point rooted maximum-likelihood phylogeny tree using 1108 core SNPs determined using reference sequence SS1 (NZ CP010867). (**c**) Phylogenetic relationship between GBS isolates belonging to CC8/CC10. Twenty-nine isolates belonged to CC8/CC10 and are represented by a mid-point rooted maximum-likelihood phylogeny tree using 2552 core SNPs called using reference sequence Sag37 (NZ CP019978.1). (**d**) Phylogenetic relationship between GBS isolates belonging to CC19. Thirty-three isolates belonged to CC19 and are represented by a mid-point rooted maximum-likelihood phylogeny tree using 2423 core SNPs called using reference sequence SG-M25 (NZ_CP021867.1). (**e**) Phylogenetic relationship between GBS isolates belonging to CC17. Thirty-two isolates belonged to CC17 and are represented by a mid-point rooted maximum-likelihood phylogeny tree using 2456 core SNPs relative to reference sequence COH1 (NZ HG9394456.1). Additional information for the isolates presented includes isolate (strain) unique identifier, capsular serotype, ST, age group (18–44 years, orange; 45–64 years, blue; 65–84 years, green; ≥85 years of age, purple; information not available, black). Where relevant, presence or absence of ARGs is shown. If none of the CC members had a particular gene present, the gene name was not added to the description column, otherwise genes were indicated as follows: tetracycline resistance gene (*tet*M, *tet*S, *tet*W, *tet*L or *tet*O gene is present, light green; absent, blank), macrolide-resistance genes (*erm*A, *erm*T, *lun*C, *lsa*C or *mef*A+*msr*D gene present, pink; absent, blank), aminoglycoside-resistance genes [*ant*(6-Ia), *aph*(3′-III), *aac*(6’)-*aph*(2’) or *aad*E gene present, salmon; absent, blank], chloramphenicol-resistance genes [*cat*Q or *cat*(pC194) gene present, black; absent, blank]. Strains indicated by an asterisk (*) highlight an uncommon combination of capsular serotype and ST. Scale bar, represents a distance of 100 point mutations.

Maximum-likelihood phylogenetic analysis has clearly shown GBS isolates falling into five main CCs (Fig. 2). The largest CC, CC23 (*n*=53), included isolates of ST23 (serotype Ia, *n*=38, and serotype III, *n*=2) and an additional seven STs of serotypes Ia and V (Fig. 3a). Serotype III ST23 isolates formed a separate clade with one isolate of serotype Ia ST1214, while serotypes Ia and V were intermixed (Fig. 3a). All CC23 isolates, bar two, carried the *tet*M gene, and only seven isolates had a combination of *tet*M and a macrolide-resistance gene (Fig. 3a). The isolates with tetracycline- and macrolide-resistance determinants did not form a cluster, suggesting random acquisition of acquired antimicrobial resistance rather than clonal expansion in the CC23 adult GBS population.

Of the 35 CC1 isolates in the study, the majority were ST1 (65.7%, *n*=23/35) and serotype V (17/23), while the remaining were serotype Ib (*n*=2), II (*n*=2) and VI (*n*=2). Ninety-one per cent (*n*=32/35) of CC1 isolates carried tetracycline-resistance genes (*tetM*, *n*=29; *tetS*, *n*=1; *tetO*, *n*=1; and *tetW*, *n*=1; Fig. 3b). Eleven CC1 GBS isolates carried *ermA* or *ermB* genes conferring resistance to macrolides, and one isolate carried additional genes conferring resistance to aminoglycosides; this isolate PHEGBS0662 (serotype IV, ST1) had *tetS*, *ermB*, *aph(3′-III*) and *ant(6-Ia*) genes and clustered closely with another serotype IV, ST1, with no acquired antimicrobial-resistance genes (ARGs) detected (Fig. 3b). This sporadic array of ARG presence is not reflective of clonal spread of resistance within the current limited dataset.

We observed two main sub-clades within CC8/CC10 (*n*=29), one of the clades was dominated by serotype Ib ST8 and another was dominated by serotypes Ib and II ST12 (Fig. 3c). All ST8 (*n*=9) isolates had *tet*M genes with one isolate also carrying the *tetO* gene, while a large sub-clade of ST12 (*n*=10) isolates carried the tetracycline-resistance gene *tet*O (Fig. 3c). Tetracycline resistance genes were common, yet diverse [*tet*(M), *tet*(O) and *tet*(L)]. Seven isolates also carried the macrolide-resistance genes *ermB* (*n*=6) and *ermA* (*n*=1). Phylogenetic separation of the isolates, bar from two isolates, PHEGBS0091 and PHEGBS0509, and the array of co-clustering ARGs is not indicative of clonal spread of resistance within the current limited dataset.

The CC19 (*n*=33) phylogeny tree showed three main clusters dominated by serotype II ST28 isolates forming a cluster together, some of the serotype III ST 19 isolates were separated in different clusters, with serotype V ST19 isolates clustering closer with serotype III ST19 isolates, PHEGBS0372 and PHEGBS0359 (Fig. 3d). The presence of tetracycline-resistance genes was almost universal (*tetM* was carried by 24 isolates and *tetO* gene was carried by 5 isolates). Eleven isolates additionally had macrolide resistance genes – *ermA* (*n*=3/33), *ermB* (*n*=6/33), *mefA+msrD* (*n*=3/33), *lnuC* (*n*=1/33) and *lsaC* (*n*=4/33) – five of these had more than one macrolide-resistance gene present (Fig. 3d). Three isolates also had additional aminoglycoside-resistance genes: *aph(3′-III*) and *ant(6-Ia*) carried by two isolates, one serotype III ST19 and one serotype V ST19; and another isolate serotype V ST19 carried the *aac(6’)-aph(2’*) gene (Fig. 3d). This was the only CC in the study to contain isolates carrying chloramphenicol-resistance genes [*n*=3 *cat*Q, *n*=1 *cat*(pC194)], which were found in serotype V (*n*=3) ST19 and III (*n*=1) ST1316 isolates (Fig. 3d).

Phylogenetic analysis of CC17 (*n*=32) indicated an intermix of the SLVs of ST17, within the serotype III ST17 isolates (Fig. 3e). Isolate ST1351 (a novel ST assigned in this study) was a serotype IV strain, not the expected serotype III, which is dominant in CC17, and additionally carried the *hvgA* hypervirulence gene, but similar non-serotype III members of CC17 have been reported elsewhere [15, 33, 40]. All isolates of CC17 carried the *tet*M gene (except for three, PHEGBS0207, PHEGBS0288 and PHEGBS0577, that carried the *tet*O gene instead), with five isolates carrying resistance genes that confer resistance to macrolides (one isolate carried *ermA* gene, two isolates carried *ermB* gene, and two isolates had both *ermB* and *mefA+msrD* genes). Four isolates had genes conferring resistance to aminoglycosides [*aadE*, *aph(3′-III*) and *ant(6-Ia*)], in addition to tetracycline and macrolide resistance genes (Fig. 3e). Overall, none of the other CCs had specific clustering observed in isolates based on geographical origin, patient age group or antimicrobial profile.

### Recombination events detected uncommon serotypes within specific genotypes

Among this collection, serotypes were associated with specific CCs, as expected (Fig. 2, Table S2). However, several exceptions were notable, where closely related strains sharing the same genotype were found to have a different capsular type (asterisks in Fig. 3), these combinations have been documented [12, 14, 16, 41], but not always investigated in detail.

**Fig. 4.**
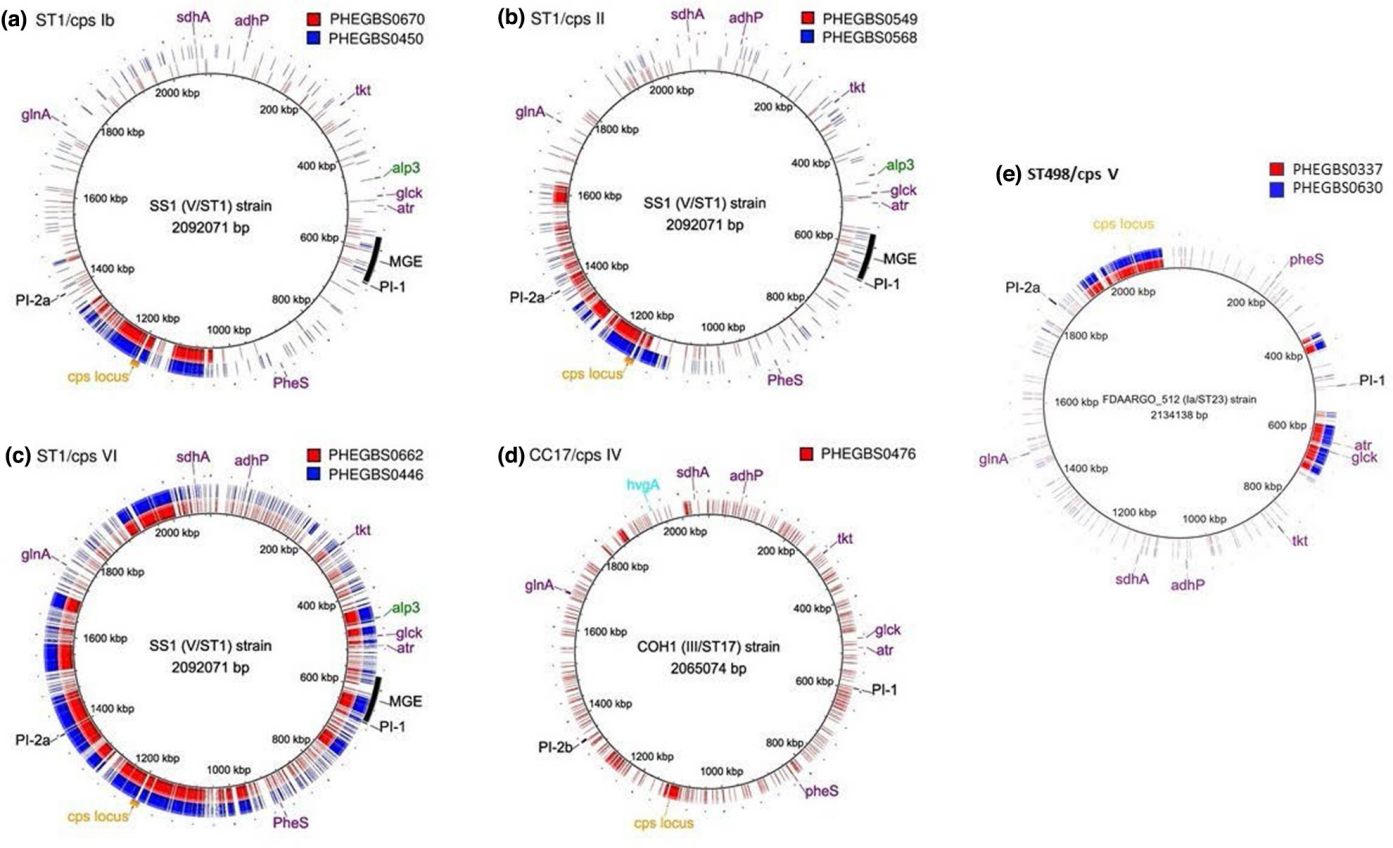
Recombination leading to serotype switching for ST1 and CC17 GBS strains from adult patients isolated from January 2014 and December 2015. Polymorphisms determined in unexpected serotypes within ST1 – serotype Ib ST1 strains PHEGBS0670 and PHEGBS0450 (**a**); serotype II ST1 strains PHEGBS0549 and PHEGBS0568 (**b**); and serotype VI ST1 strains PHEGBS0662 and PHEGBS0446 (**c**) are plotted against the reference serotype V ST1 strain SS1 (NZ_CP010867). Serotype IV ST1351 (CC17) PHEGBS0476 strain (**d**) is plotted against serotype III ST17 reference strain COH1 (NZ_HG9394456.1). Serotype V CC23-ST498 isolates PHEGBS0337 and PHEGBS0630 (e) are plotted against ST23 serotype Ia reference strain FDAARGOS_512 (NZ_CP033822.1). The innermost (black circle) represents the respective reference SS1, COH1 or FDAARGO_512. The following genome landmarks are shown in the outermost circle: cps locus, capsular polysaccharide locus, in orange; *alp*3, alpha-like surface protein-encoding gene in green; *hvgA*, hypervirulent surface-anchored adhesin gene in aqua; seven MLST genes (*adhP, atr, tkt, glcK, sdhA, glnA* and *pheS*) in purple; MGE, mobile genetic element, in black; *PI-1* and *PI-2a*, pilus island genes also in black. Polymorphisms mapping to MGE identified in the various non–serotype V ST1 strains were not included in the analysis.

Six isolates of the ST1 lineage were identified in this study that carried a ST1 core genome that is more consistent with serotype V but show a potential capsular switching event with serotypes Ib, II and VI (Fig. 4). PHEGBS0670 and PHEGBS0450 (both serotype Ib, ST1) acquired DNA regions of 217 and 209 kb, respectively, including the *cps*Ib locus (Fig. 4a); PHEGBS0549 and PHEGBS0568 (both serotype II) acquired DNA regions of 232 and 144 kb, respectively, including the *cps*II locus (Fig. 4b). In contrast, serotype VI ST1 isolates PHEGBS0662 and PHEGBS0446 had larger-scale recombination events (including the *cps*IV locus) of 883 and 884 kb, respectively, involving approximately 45% of the genomes, with the resultant genomes containing all of the ST1 MLST determinants (Fig. 4c); similar large-scale recombination events of ST1 isolates have previously led to the conclusion that these are unlikely to have been capsular switching events [12], which is applicable to the two serotype VI ST1 isolates reported in our study as well. Otherwise, all six isolates retained high similarity to reference strain SS1 and all contained the *alp*3 gene and *PI-1* and *PI-2a* pilus combination common to serotype V strains [42, 43]. To confirm the identified capsular switching events, ST1 isolates in this study were compared to previously published ST1 isolates with recombination events: ST1-serotype Ib (SH5446) from Portugal [32], and ST1-serotype II (NGBS748) and ST1-serotype VI (NGBS209) from Canada [12]. Phylogenetic analysis of these three GBS isolates with ST1 isolates in this study showed dispersal throughout the UK of the ST1 phylogenetic cluster, with a mean of 207 and 583 SNPs per serotype Ib and VI ST1 isolate, respectively (Fig. S1). The recombination borders were nearly identical for all isolates, confirming the potential capsular switching events have taken place between serotypes V and Ib and serotype V and II (Fig. S2a).

On examination of the potential capsular switch event in serotype IV isolate ST1351 within CC17 (ST1351 is a SLV of ST17), this isolate carried *hvgA*, which is commonly associated with hypervirulent ST17 strains. Recombination analysis indicated a PHEGBS0476 potential for capsular switching could be attributed to an event with a 31.3 kb DNA fragment exchange that contained the entire *cps*IV operon, comparable to the DNA transfer of 35.5 kb reported in a serotype IV/ST17 isolate from France (CC209361) [16] (Figs 4d and S3). Comparison of this isolate to other IV isolates from CC1 revealed the potential transfer of *cpsE-cpsG* and *neuB-neuA* genes was highly conserved, irrespective of whether they were present in isolates with determinants designating them CC1 or CC17, not only in isolates from this study, but also in isolates previously reported to undergo capsular switching in CC1 and CC17 serotype IV isolates from France (CC209361 [16]) and Ireland (GBS148, GBS150 and GBS175 [44]), where two SNPs, one in each of the *cpsE* and *cpsF* genes (PHEGBS0476) were observed (Fig. S3).

We have noted that three ST498 (CC23) isolates were serotypes Ia (*n*=1, most common serotype within CC23) and V (*n*=2). Further genomic investigation of the two serotype V isolates identified large recombination blocks (353 kb in isolate PHEGBS0337 and 352 kb in PHEGBS0630) that were spanning across the whole capsular locus, indicating a potential capsular switch between serotype Ia and V (Fig. 4e). We investigated potential recombination events between five serotype V ST19 isolates (PHEGBS139, PHEGBS595, PHEGBS608, PHEGBS657 and PHEGBS738), which formed a cluster in the CC19 clade (Fig. 3d). These isolates were compared to a serotype III ST19 reference. Recombinant DNA regions identified included the *cps*V locus in PHEGBS139, PHEGBS595, PHEGBS608 of 301 kb in size, and in PHEGBS657 and PHEGBS738 acquisition of 334 kb and 344 kb regions, respectively (Fig. S2b). We have not detected any of the transposons present in close vicinity of the identified recombination blocks within the capsular region in any of the isolates analysed in this study.

## Discussion

Previous reports noted an increase of GBS infections in adults between 1991–2010 and 2015–2016 in England and Wales [3, 5]. Our study demonstrates the serotype and genotype distribution of GBS infections reported in adults between 2014 and 2015 in the UK. Serotypes III, Ia and V predominate in this data set, indicating no change in the three predominant serotypes the UK population since 2010 [3]. Despite this, a high genomic diversity has been identified with 47 different MLST genotypes determined, including 15 new STs, all clustering into five major clonal clusters (CC1, CC8, CC17, CC19 and CC23) as expected. Of importance, no geographical nor phylogenetic clusters of isolates were noted, indicating that in this limited dataset evidence of transmission events was not found.

In this study, we have noted four potential capsular switching events involving isolates of ST1, CC17, CC23 and ST19 genotypes. Recombination leading to capsular switching involved isolates exhibiting serotypes Ib (*n*=2, both ST1 genetic background), II (*n*=2, ST1 genetic background), IV (*n*=1, ST1351/CC17 genetic background) and V (*n*=7, 2 isolates of ST498/CC23 and 5 isolates of ST19 genetic background). Interestingly, ST1 serotypes Ib and II isolates did not cluster together in the phylogenetic tree after recombination removal, suggesting distinct recombination events within the ST1 GBS population. For the ST498 and ST19 isolates, with respective genotype backgrounds serotype Ia and III, recombination resulted in co-clustered serotype V isolates in each of the lineages during phylogenetic analysis, suggesting a common or single capsular switching event to serotype V with expansion.

Some of the capsular switching events identified in this study have been previously reported in Portugal [32], Canada [12] and France [16]. However, to the best of our knowledge, investigations into recombination for CC23 and ST19 isolates have not been reported. We have observed novel capsular switching events in ST1 isolates, with isolates of serotype Ib and II showing recombined capsular regions compared to the reference sequence of serotype V ST1, confirming findings from a previous study [12]. CC17 isolates have been reported to be mostly clonal and only one capsular switching event to serotype IV [16] has been reported to date, which we observed also in a ST1351 (CC17) serotype IV isolate. With increasing studies pertaining to GBS genomic epidemiology, we gain further understanding of the diversity of serotype–genotype combinations, the spread of AMR determinants within GBS populations, the global distribution of clones of interest, their associations with specific host populations, and disease or carriage status.

Genes encoding the most abundant GBS surface Alp family proteins, such as Alp2, Alp3, Alpha C (*bca*) and Rib, were readily identified in this GBS dataset. Alpha C protein in GBS is important for interactions with cervical and epithelial cells to promote intracellular invasion and dissemination [45, 46]. The other Alp family surface proteins are also of great interest as they provide immunological reaction and potentially protection against GBS disease [47]. Due to this, studies report associations observed between GBS serotypes and genotypes. A recent meta-analysis study on global GBS isolates has reported that 79% of invasive adult GBS had at least one of the Alp2, Alp3, Alpha C (*bca*) or Rib protein genes [6]; a similar distribution has been observed in our study as well, with 72.5% (*n*=140/193) of the isolates carrying one of the mentioned Alp family proteins. We have found serotype III isolates were reported to be more commonly associated with Rib protein and serotype V isolates to be associated with Alp3, which concurs with previous reports [6]; and the Alpha C protein (*bca* gene) was found to be associated with serotype Ib in our study, though it has been reported to be associated with serotypes Ib and Ia [6].

Due to their antigenic properties, Alp family proteins have been proposed as potential targets for protein-based GBS vaccines, one such vaccine using the N-terminal domain of AlphaC and Rib has been studied in Clinical Trials Phase I (NCT02459262) already. Vaccines targeting GBS pilus proteins have been also proposed, considering that these proteins are present universally in GBS strains, with *PI-1+PI-2a* being the most common [6], and were identified in this study also. However, there is a lack of data supporting their potential as vaccine candidates at this time and further analysis is needed [48, 49].

This study has several limitations; the sample set is a cross-sectional observational study limited to only referred isolates within adults over the time period in question. It does not represent an accurate number of isolates from cases with disease burden nor include isolates from screening adults. However, though this dataset is not representative of all GBS infection in adults during the study period, isolates analysed here are likely to be representative of infection of greatest severity, which are more likely to be sent to the reference laboratory. Irrespective, this study highlights the need for continuous genomic surveillance to monitor genotype, genotypic capsular serotype, and genes associated with hypervirulence and antibiotic resistance to help inform future treatment of cases and potential vaccination programmes, including polysaccharide- or protein-based GBS vaccines.

## Supplementary Data

Supplementary material 1
