# Supplementary material for: Identifying large-scale recombination and capsular switching events in Streptococcus agalactiae strains causing disease in adults in the UK between 2014 and 2015
Source: Microb Genom. 2022 Mar 15;8(3):000783. doi: 10.1099/mgen.0.000783 (PMC9176283; doi:10.1099/mgen.0.000783)
Supplement: Supplementary material 1 [file mgen-8-0783-s001.pdf]

Supplementary Information

Identifying large-scale recombination and capsular switching events in *Streptococcus agalactiae* strains causing disease in adults in the United Kingdom between 2014 and 2015

Uzma Basit Khan[1,a]*, Elita Jauneikaite[2,3]*, Robert Andrews[4], Victoria J Chalker[5] and Owen B. Spiller[1,5#]

Supplementary Tables

Tables S1. Assembly statistics for 193 whole genome sequences used in this study. Assembly statistics taken from QUAST analysis, all results reported are for contigs >=500bp.

Sample ID	ERA number	Assembly accession number	Assembled genome length	No. of contigs	length of largest contig (bp)	N50 (bp)
100414	ERR2560245	GCA_920937375	2359530	185	322105	109165
141439	ERR3531632	GCA_920937465	2025404	36	282949	111138
200682	ERR3531626	GCA_920937515	2235218	68	168472	79621
200683	ERR3531633	GCA_920937495	2048139	18	363701	213107
200684	ERR3531637	GCA_920937505	2055521	24	286179	121246
200690	ERR3531636	GCA_920937415	2042714	21	456519	179336
200693	ERR3531627	GCA_920937345	1964400	35	332173	92076
200694	ERR3531635	GCA_920937355	2076003	35	387800	94235
200696	ERR3531628	GCA_920937385	2013400	33	336548	92899
200697	ERR3531629	GCA_920937525	2029229	32	294450	93048
200698	ERR3531634	GCA_920937405	2061821	19	501932	275800
200699	ERR3531631	GCA_920937475	2113316	37	261420	80353
200702	ERR3531630	GCA_920937925	1999895	18	825426	263403
200706	ERR3531625	GCA_920937395	2052960	20	368886	209879
200710	ERR3589625	GCA_920937535	2022921	27	260511	142987
PHEGBS0041	ERR1742042	GCA_920937445	2055687	29	345110	110270
PHEGBS0042	ERR1742087	GCA_920937425	1993860	35	261499	140419
PHEGBS0044	ERR1742051	GCA_920937455	2080855	31	372108	115460
PHEGBS0047	ERR1741500	GCA_920937435	1995537	24	422800	123141
PHEGBS0048	ERR1741806	GCA_920937555	2064119	20	524213	152327
PHEGBS0049	ERR1741385	GCA_920937775	2014596	34	267633	135263
PHEGBS0052	ERR1741861	GCA_920937715	1986454	34	271787	92359
PHEGBS0054	ERR1742070	GCA_920937655	2035643	22	500651	152745
PHEGBS0060	ERR1741459	GCA_920984595	2067203	25	465676	145542
PHEGBS0061	ERR1741474	GCA_920937935	2121892	40	239669	115559
PHEGBS0066	ERR1741366	GCA_920937675	2005768	35	249543	124078
PHEGBS0067	ERR1741677	GCA_920937565	2074018	27	541324	129106
PHEGBS0068	ERR1741595	GCA_920937575	2181140	35	537406	170965
PHEGBS0070	ERR1742116	GCA_920938025	1985181	26	384866	195246

PHEGBS0071	ERR1742139	GCA_920937975	2115399	69	271485	56306
PHEGBS0072	ERR1741514	GCA_920937585	2068618	47	383569	90189
PHEGBS0080	ERR1741859	GCA_920937705	2032633	88	92771	42988
PHEGBS0081	ERR1741497	GCA_920938285	2052375	22	344927	179301
PHEGBS0082	ERR1741501	GCA_920937795	2066202	22	623892	173759
PHEGBS0084	ERR1741695	GCA_920937595	2162014	22	427702	131081
PHEGBS0086	ERR1741926	GCA_920937485	2047057	19	566092	468085
PHEGBS0090	ERR1742102	GCA_920937735	2133485	25	555418	119351
PHEGBS0091	ERR1741407	GCA_920937725	2192871	31	285443	150407
PHEGBS0092	ERR1741722	GCA_920937945	2162627	45	188493	103768
PHEGBS0095	ERR1741918	GCA_920937615	2028040	21	649906	195022
PHEGBS0097	ERR1741888	GCA_920937635	2157776	30	436280	109113
PHEGBS0098	ERR1741573	GCA_920937955	2114109	21	345272	120163
PHEGBS0100	ERR1741744	GCA_920937785	2079895	58	142136	68038
PHEGBS0106	ERR1742039	GCA_920937905	2006154	21	422759	151565
PHEGBS0117	ERR1741635	GCA_920937755	1996845	27	280548	124174
PHEGBS0121	ERR1741801	GCA_920937875	2020928	19	509200	120015
PHEGBS0122	ERR1741539	GCA_920937605	2014362	16	649976	215508
PHEGBS0123	ERR1742074	GCA_920937625	2027693	23	409959	137831
PHEGBS0127	ERR1741367	GCA_920937895	2055945	25	522317	125962
PHEGBS0128	ERR1742140	GCA_920937685	2058985	23	354729	159640
PHEGBS0132	ERR1741466	GCA_920938305	2248273	55	321926	74630
PHEGBS0134	ERR1741692	GCA_920937885	1960272	16	604929	240851
PHEGBS0135	ERR1741673	GCA_920937915	2111991	25	364461	137333
PHEGBS0139	ERR1741954	GCA_920937545	2085983	28	264960	129372
PHEGBS0144	ERR1741588	GCA_920937805	2034729	34	264205	92386
PHEGBS0145	ERR1741818	GCA_920937965	2128399	20	397935	157535
PHEGBS0151	ERR1741944	GCA_920937855	1981843	28	275506	114049
PHEGBS0152	ERR1742041	GCA_920937845	2041947	19	482420	263243
PHEGBS0153	ERR1741822	GCA_920937665	2050273	35	513939	121803
PHEGBS0154	ERR1741575	GCA_920937695	2031222	21	363662	213798
PHEGBS0156	ERR1741857	GCA_920937765	2149526	37	251765	116606
PHEGBS0164	ERR1741749	GCA_920937745	2053728	20	345271	190986
PHEGBS0170	ERR1742029	GCA_920937645	2013304	18	389473	151886
PHEGBS0171	ERR1741963	GCA_920937865	2131791	39	188287	82359
PHEGBS0176	ERR1742030	GCA_920938325	2055010	14	557767	233672
PHEGBS0188	ERR1741906	GCA_920938145	1995197	18	688163	138887
PHEGBS0193	ERR1742119	GCA_920937985	2039304	78	488568	139079
PHEGBS0194	ERR1741932	GCA_920984575	2094588	58	146249	53681
PHEGBS0206	ERR1741687	GCA_920938365	2166025	20	698963	199321
PHEGBS0207	ERR1741997	GCA_920938415	2102437	30	311394	161585
PHEGBS0219	ERR1741590	GCA_920938015	2014180	33	385922	114716
PHEGBS0230	ERR1741957	GCA_920938075	2000018	15	788662	252592
PHEGBS0237	ERR1742117	GCA_920938195	2062447	31	280309	99729
PHEGBS0243	ERR1741633	GCA_920938115	2043562	33	280454	92994
PHEGBS0246	ERR1741679	GCA_920938655	1968977	23	619252	176684
PHEGBS0248	ERR1742038	GCA_920938615	2042767	26	522377	145462
PHEGBS0252	ERR1741658	GCA_920938395	2104508	39	217813	99224
PHEGBS0253	ERR1741828	GCA_920938095	2116303	25	343732	133515
PHEGBS0265	ERR1741375	GCA_920938065	2032574	17	505921	164379
PHEGBS0266	ERR1741584	GCA_920938335	2158950	25	380390	135560
PHEGBS0267	ERR1741548	GCA_920938625	2014775	27	524006	105548
PHEGBS0270	ERR1742082	GCA_920938105	2032512	20	699553	158859
PHEGBS0275	ERR1742034	GCA_920937995	2043269	32	260499	91995
PHEGBS0283	ERR1741523	GCA_920938235	2041895	31	680187	102997

PHEGBS0286	ERR1741880	GCA_920938635	2102353	34	243834	102257
PHEGBS0287	ERR1742120	GCA_920938355	2022675	23	519633	132699
PHEGBS0288	ERR1741473	GCA_920938385	2159710	33	285123	111616
PHEGBS0295	ERR1741889	GCA_920938165	1965311	16	383540	221142
PHEGBS0296	ERR1741462	GCA_920938175	2141322	53	187402	63995
PHEGBS0300	ERR1741553	GCA_920938505	2041257	28	219972	115462
PHEGBS0306	ERR1741485	GCA_920938535	2090086	21	341391	191063
PHEGBS0308	ERR1741488	GCA_920938515	2196708	34	198658	107110
PHEGBS0318	ERR1741792	GCA_920938205	1999622	30	235807	92560
PHEGBS0320	ERR1741605	GCA_920938125	2119360	64	147168	48327
PHEGBS0336	ERR1741747	GCA_920938445	2014037	23	442764	149380
PHEGBS0337	ERR1741620	GCA_920938525	2088706	16	456155	299098
PHEGBS0355	ERR1741387	GCA_920938545	2010507	41	440120	92091
PHEGBS0359	ERR1741657	GCA_920938215	2121307	42	269427	96654
PHEGBS0360	ERR1742032	GCA_920938035	2053822	35	301380	76544
PHEGBS0367	ERR1742111	GCA_920938575	2014630	18	545026	155385
PHEGBS0368	ERR1742016	GCA_920938565	2054477	22	536362	119351
PHEGBS0372	ERR1741524	GCA_920938055	2141436	36	236840	95233
PHEGBS0373	ERR1741478	GCA_920938455	2054315	21	310516	141491
PHEGBS0377	ERR1741496	GCA_920938465	2012185	33	258776	91170
PHEGBS0378	ERR1741669	GCA_920938585	2104768	29	340979	114468
PHEGBS0380	ERR1741867	GCA_920938045	2026201	36	280827	94183
PHEGBS0382	ERR1741847	GCA_920938155	2060450	35	253225	118805
PHEGBS0383	ERR1741449	GCA_920938135	2038822	36	235651	91860
PHEGBS0389	ERR1741939	GCA_920938085	2013939	41	277651	87763
PHEGBS0390	ERR1741684	GCA_920938475	2229466	30	235526	104999
PHEGBS0393	ERR1741475	GCA_920938605	2141907	30	243431	157535
PHEGBS0394	ERR1741769	GCA_920938485	2089389	27	358893	148355
PHEGBS0398	ERR1741711	GCA_920938295	1988018	81	122438	42295
PHEGBS0399	ERR1741537	GCA_920938345	2104536	29	321549	84885
PHEGBS0401	ERR1741671	GCA_920938375	2028548	24	389536	152780
PHEGBS0407	ERR1741602	GCA_920938555	2037136	22	497031	118979
PHEGBS0408	ERR1741526	GCA_920938405	2107102	28	553837	97162
PHEGBS0411	ERR1742019	GCA_920938425	2132584	34	255247	113252
PHEGBS0416	ERR1741402	GCA_920938645	2001227	32	280573	95462
PHEGBS0428	ERR1741389	GCA_920938435	2120073	27	282551	147580
PHEGBS0429	ERR1741515	GCA_920938185	1995933	32	280722	120214
PHEGBS0446	ERR1741823	GCA_920938005	2100525	25	393662	169307
PHEGBS0447	ERR1741785	GCA_920938275	2007286	14	712860	136803
PHEGBS0448	ERR1741564	GCA_920938595	2102878	37	322662	153339
PHEGBS0450	ERR1741493	GCA_920938255	2048737	18	341394	191113
PHEGBS0463	ERR1742031	GCA_920938245	2152913	40	241901	95189
PHEGBS0464	ERR1741702	GCA_920938265	2073518	24	323754	193184
PHEGBS0465	ERR1741442	GCA_920938225	2005280	19	526411	152096
PHEGBS0467	ERR1741981	GCA_920938495	2369887	182	153218	32888
PHEGBS0476	ERR1741458	GCA_920938315	2079208	44	344370	94690
PHEGBS0480	ERR1741862	GCA_920938785	2048236	34	240044	92967
PHEGBS0483	ERR1741662	GCA_920939095	1961899	62	155038	47762
PHEGBS0491	ERR1741535	GCA_920939115	2046271	33	340400	117554
PHEGBS0492	ERR1741922	GCA_920938755	2185096	41	250829	115984
PHEGBS0493	ERR1741913	GCA_920938775	2058597	26	341389	115567
PHEGBS0501	ERR1741911	GCA_920938795	2003048	36	331270	91861
PHEGBS0503	ERR1741579	GCA_920939045	2074653	51	181045	83817
PHEGBS0509	ERR1741644	GCA_920939235	2152255	41	216372	122905
PHEGBS0512	ERR1741958	GCA_920938765	1950594	20	493601	141044

PHEGBS0513	ERR1741701	GCA_920938705	2022171	36	203067	92203
PHEGBS0518	ERR1741606	GCA_920939075	2096669	13	676614	471088
PHEGBS0520	ERR1741361	GCA_920938915	2122550	20	426683	153584
PHEGBS0524	ERR1741696	GCA_920939125	2037514	14	454928	230800
PHEGBS0527	ERR1742059	GCA_920939085	2018034	18	497655	263402
PHEGBS0532	ERR1741494	GCA_920939105	2092344	20	481015	290420
PHEGBS0533	ERR1741445	GCA_920938735	2067865	21	345357	155748
PHEGBS0539	ERR1741599	GCA_920938695	2065877	25	326493	139677
PHEGBS0547	ERR1741621	GCA_920938725	2019045	16	712625	124094
PHEGBS0549	ERR1741850	GCA_920939225	2049432	23	442401	114929
PHEGBS0551	ERR1741634	GCA_920939055	2063747	21	320648	141173
PHEGBS0552	ERR1741992	GCA_920939065	2068680	33	211892	152944
PHEGBS0554	ERR1741388	GCA_920938825	2170408	20	358371	201882
PHEGBS0555	ERR1741852	GCA_920938715	2038087	28	369057	114835
PHEGBS0556	ERR1741948	GCA_920984555	2065555	18	321233	211798
PHEGBS0559	ERR1741492	GCA_920984565	2104667	39	260697	91861
PHEGBS0561	ERR1741542	GCA_920984585	2042133	57	512647	91862
PHEGBS0566	ERR1741860	GCA_920939025	2110525	41	188728	102075
PHEGBS0567	ERR1741438	GCA_920938945	2146678	42	212845	83528
PHEGBS0568	ERR1741647	GCA_920938665	2077020	25	322975	121677
PHEGBS0575	ERR1741521	GCA_920938885	2156314	30	205589	113105
PHEGBS0577	ERR1741835	GCA_920939035	2156314	30	205589	113105
PHEGBS0581	ERR1741660	GCA_920938925	2196603	32	299937	98941
PHEGBS0586	ERR1741614	GCA_920939215	2014066	31	245811	96789
PHEGBS0589	ERR1741794	GCA_920938835	1997662	26	384071	102978
PHEGBS0592	ERR1741448	GCA_920939185	2061511	36	187233	83449
PHEGBS0593	ERR1741754	GCA_920939145	2133734	61	195821	79027
PHEGBS0595	ERR1741483	GCA_920939165	2109564	43	261494	110912
PHEGBS0598	ERR1741680	GCA_920938955	2191974	144	226421	78419
PHEGBS0599	ERR1741659	GCA_920938805	2087564	40	240216	96266
PHEGBS0608	ERR1741534	GCA_920939245	2193800	42	222835	78745
PHEGBS0610	ERR1742130	GCA_920938815	2018455	40	268329	91995
PHEGBS0616	ERR1742012	GCA_920939005	2054548	23	264386	132697
PHEGBS0618	ERR1741842	GCA_920939175	2037557	18	509111	315905
PHEGBS0622	ERR1741630	GCA_920938745	2116739	20	282488	162699
PHEGBS0623	ERR1741456	GCA_920938985	2002455	16	610192	146436
PHEGBS0624	ERR1741580	GCA_920939195	2128528	23	422783	193178
PHEGBS0625	ERR1742108	GCA_920939205	2021983	26	332982	123463
PHEGBS0626	ERR1741728	GCA_920938875	2093089	30	341537	144721
PHEGBS0627	ERR1741512	GCA_920938995	2108400	25	517430	138034
PHEGBS0630	ERR1741525	GCA_920938855	2086654	19	345179	182496
PHEGBS0635	ERR1741616	GCA_920938965	2158160	13	647134	231450
PHEGBS0639	ERR1741887	GCA_920939015	2146414	40	191783	79040
PHEGBS0643	ERR1741746	GCA_920938675	2146187	28	300200	116921
PHEGBS0648	ERR1741752	GCA_920938935	2052740	18	374106	180784
PHEGBS0654	ERR1741549	GCA_920938845	2002532	17	610122	156485
PHEGBS0657	ERR1741517	GCA_920938975	2081054	35	282212	104675
PHEGBS0658	ERR1741901	GCA_920938865	1977354	21	672968	135323
PHEGBS0662	ERR1741427	GCA_920938895	2179091	48	483415	97514
PHEGBS0663	ERR1741436	GCA_920938685	2103888	23	533819	149710
PHEGBS0664	ERR1742011	GCA_920938905	2126065	35	322486	89438
PHEGBS0667	ERR1741656	GCA_920939255	2079376	20	340964	157718
PHEGBS0670	ERR1742049	GCA_920939135	2117572	20	687499	169060
PHEGBS0738	ERR1741902	GCA_920939155	2126457	99	151596	48064

Table S2. Summary of characteristics of 193 GBS whole genome sequences used in this study. During 20 months of surveillance between January 2014 and December 2015, a total of 193 GBS isolates causing disease in adults were whole genome sequenced. Isolates were grouped into invasive and non-invasive disease-causing GBS based on the site of isolates (specimen column); isolates from blood (n=178), CSF (n=1), aortic valve (n=1), amniotic membrane (n=1), placenta (n=3) were classed as invasive; and pus (n=2), throat swab (n=1), tissue (n=2), vaginal swab (n=1), wound swab (n=1) and abscess (n=1) were classed as non-invasive; one isolates did not have information where it was isolated from. Surface (alpha and beta like) proteins, *alp2, alp3, bca, cba, rib*, bacterial adhesin genes, *bibA* and *hvgA*, were detected. Novel STs identified in this study are marked in bold. *uncertainty detected for reported ST for this isolate; # - clonal cluster was reassigned based on clustering from phylogenetic analysis. F- female, M – male, n/a – information not available.

Sample ID	ERA number	Serotype	ST	CC_MLST	CC_wgs#	*alp2*	*alp3*	*bca*	*cba*	*rib*	*bib*A	*hvgA*	Pilus island genes	Sex	Age group	Specimen	Region
PHEGBS0447	ERR1741785	Ia	ST1065	CC23	CC23	no	no	no	no	no	yes	no	*PI-1+PI+2a*	F	18-44	Blood	Greater London
PHEGBS0618	ERR1741842	Ia	**ST1214**	singleton	CC23	yes	no	no	no	no	no	no	*PI-1+PI+2a*	F	65-84	Blood	Yorkshire and the Humber
PHEGBS0134	ERR1741692	Ia	**ST1218**	CC23	CC23	no	no	no	no	no	yes	no	*PI-1+PI-2a*	M	45-64	Blood	Yorkshire and the Humber
PHEGBS0616	ERR1742012	Ia	ST24	CC498/CC24	CC23	no	no	yes	no	no	yes	no	*PI-1+PI-2a*	F	18-44	Blood	Wales
PHEGBS0188	ERR1741906	Ia	ST24	CC498/CC24	CC23	no	no	yes	no	no	yes	no	*PI-1+PI-2a*	F	18-44	Blood	Wales
PHEGBS0086	ERR1741926	Ia	**ST1317**	CC23	CC23	no	no	no	no	no	yes	no	*PI-1+PI-2a*	M	45-64	Wound swab	South East England
PHEGBS0663	ERR1741436	Ia	ST144	CC23	CC23	no	no	no	no	yes	yes	no	*PI-1+PI-2a*	F	18-44	Blood	Greater London
PHEGBS0049	ERR1741385	Ia	ST144	CC23	CC23	no	no	no	no	yes	yes	no	*PI-1+PI-2a*	M	85+	Blood	North East England
PHEGBS0248	ERR1742038	Ia	ST23	CC23	CC23	no	no	no	no	no	yes	no	*PI-1+PI-2a*	F	18-44	Blood	South East England
PHEGBS0194	ERR1741932	Ia	ST7	singleton	CC8/CC10	no	no	yes	yes	no	no	no	*PI-1+PI-2b*	M	85+	Blood	South East England
PHEGBS0230	ERR1741957	Ia	ST23	CC23	CC23	no	no	no	no	no	yes	no	*PI-1+PI-2a*	F	85+	Blood	South East England
PHEGBS0044	ERR1742051	Ia	ST23	CC23	CC23	no	no	no	no	no	yes	no	*PI-1+PI-2a*	F	18-44	Blood	Northern Ireland
PHEGBS0047	ERR1741500	Ia	ST24	CC498/CC24	CC23	no	no	yes	no	no	yes	no	*PI-1+PI-2a*	M	45-64	Blood	South West England
PHEGBS0547	ERR1741621	Ia	ST23	CC23	CC23	no	no	no	no	no	yes	no	*PI-1+PI-2a*	F	85+	Blood	Wales
PHEGBS0170	ERR1742029	Ia	ST23	CC23	CC23	no	no	no	no	no	yes	no	*PI-1+PI-2a*	F	85+	Blood	North West England
PHEGBS0503	ERR1741579	Ia	ST23	CC23	CC23	no	no	no	no	no	yes	no	*PI-1+PI-2a*	M	65-84	Blood	South East England
PHEGBS0320	ERR1741605	Ia	ST7	singleton	CC8/CC10	no	no	yes	yes	no	no	no	*PI-1+PI-2b*	F	65-84	Blood	Yorkshire and the Humber
PHEGBS0070	ERR1742116	Ia	ST23	CC23	CC23	no	no	no	no	no	yes	no	*PI-1+PI-2a*	F	65-84	Blood	South East England
PHEGBS0060	ERR1741459	Ia	ST23	CC23	CC23	no	no	no	no	no	yes	no	*PI-1+PI-2a*	F	85+	Blood	South East England
PHEGBS0283	ERR1741523	Ia	ST23	CC23	CC23	no	no	no	no	no	yes	no	*PI-1+PI-2a*	F	18-44	Blood	South East England
PHEGBS0048	ERR1741806	Ia	ST23	CC23	CC23	no	no	no	no	no	yes	no	*PI-1+PI-2a*	M	65-84	Blood	South West England
PHEGBS0193	ERR1742119	Ia	ST23	CC23	CC23	no	no	yes	no	yes	yes	no	*PI-1+PI-2a*	F	18-44	Blood	Scotland
PHEGBS0527	ERR1742059	Ia	ST23	CC23	CC23	no	no	no	no	no	yes	no	*PI-1+PI-2a*	F	18-44	Blood	Greater London
PHEGBS0300	ERR1741553	Ia	ST23	CC23	CC23	no	no	no	no	no	yes	no	*PI-1+PI-2a*	F	18-44	Blood	Greater London
PHEGBS0512	ERR1741958	Ia	ST23	CC23	CC23	no	no	no	no	no	yes	no	*PI-1+PI-2a*	F	18-44	Blood	Greater London
PHEGBS0265	ERR1741375	Ia	ST23	CC23	CC23	no	no	no	no	no	yes	no	*PI-1+PI-2a*	F	18-44	Blood	Greater London
200702	ERR3531630	Ia	ST23	CC23	CC23	no	no	no	no	no	yes	no	*PI-1+PI-2a*	M	45-64	Blood	North West England
PHEGBS0123	ERR1742074	Ia	ST23	CC23	CC23	no	no	no	no	no	yes	no	*PI-1+PI-2a*	F	18-44	Blood	Greater London
PHEGBS0465	ERR1741442	Ia	ST23	CC23	CC23	no	no	no	no	no	yes	no	*PI-1+PI-2a*	F	18-44	Aortic valve	Greater London
PHEGBS0407	ERR1741602	Ia	ST23	CC23	CC23	no	no	no	no	no	yes	no	*PI-1+PI-2a*	F	45-64	Blood	Greater London
PHEGBS0121	ERR1741801	Ia	ST23	CC23	CC23	no	no	no	no	no	yes	no	*PI-1+PI-2a*	F	18-44	Blood	East England
PHEGBS0539	ERR1741599	Ia	ST23	CC23	CC23	no	no	no	no	no	yes	no	*PI-1+PI-2a*	F	18-44	Amniotic membrane	Wales
PHEGBS0106	ERR1742039	Ia	ST23	CC23	CC23	no	no	no	no	no	yes	no	*PI-1+PI-2a*	F	18-44	Blood	South East England
PHEGBS0095	ERR1741918	Ia	ST23	CC23	CC23	no	no	no	no	no	yes	no	*PI-1+PI-2a*	F	45-64	Blood	South West England
PHEGBS0623	ERR1741456	Ia	ST23	CC23	CC23	no	no	no	no	no	yes	no	*PI-1+PI-2a*	F	65-84	Blood	South East England
PHEGBS0654	ERR1741549	Ia	ST23	CC23	CC23	no	no	no	no	no	yes	no	*PI-1+PI-2a*	F	65-84	Blood	South East England
PHEGBS0295	ERR1741889	Ia	ST23	CC23	CC23	no	no	no	no	no	yes	no	*PI-1+PI-2a*	F	18-44	Blood	North East England
PHEGBS0267	ERR1741548	Ia	ST23	CC23	CC23	no	no	no	no	no	yes	no	*PI-1+PI-2a*	M	65-84	Tissue	Scotland
PHEGBS0401	ERR1741671	Ia	ST23	CC23	CC23	no	no	no	no	no	yes	no	*PI-1+PI-2a*	M	65-84	Blood	East Midlands
PHEGBS0246	ERR1741679	Ia	ST23	CC23	CC23	no	no	no	no	no	yes	no	*PI-1+PI-2a*	M	65-84	Blood	Yorkshire and the Humber
PHEGBS0270	ERR1742082	Ia	ST23	CC23	CC23	no	no	no	no	no	yes	no	*PI-1+PI-2a*	M	65-84	Blood	Yorkshire and the Humber
PHEGBS0625	ERR1742108	Ia	ST23	CC23	CC23	no	no	no	no	no	yes	no	*PI-1+PI-2a*	M	65-84	Blood	Yorkshire and the Humber
PHEGBS0067	ERR1741677	Ia	ST23	CC23	CC23	no	no	no	no	no	yes	no	*PI-1+PI-2a*	M	18-44	Blood	East England
PHEGBS0152	ERR1742041	Ia	ST24	CC498/CC24	CC23	no	no	yes	no	no	yes	no	*PI-1+PI-2a*	M	65-84	Blood	North West England
PHEGBS0589	ERR1741794	Ia	ST23	CC23	CC23	no	no	no	no	no	yes	no	*PI-1+PI-2a*	M	65-84	Blood	East England
PHEGBS0336	ERR1741747	Ia	ST23	CC23	CC23	no	no	no	no	no	yes	no	*PI-1+PI-2a*	M	65-84	Blood	South East England

Table S2 (continued)

Sample ID	ERA number	Serotype	ST	CC_MLST	CC_wgs#	*alp2*	*alp3*	*bca*	*cba*	*rib*	*bibA*	*hvgA*	Pilus island genes	Sex	Age group	Specimen	Region
PHEGBS0367	ERR1742111	Ia	ST23	CC23	CC23	no	no	no	no	no	yes	no	*PI-1+PI-2a*	M	65-84	Blood	South East England
PHEGBS0518	ERR1741606	Ia	ST498	CC498/CC24	CC23	no	no	yes	no	no	yes	no	*PI-1+PI-2a*	F	18-44	Blood	East England
PHEGBS0658	ERR1741901	Ia	ST23	CC23	CC23	no	no	no	no	no	yes	no	*PI-1+PI-2a*	M	65-84	Blood	South East England
PHEGBS0287	ERR1742120	Ia	ST23	CC23	CC23	no	no	no	no	no	yes	no	*PI-1+PI-2a*	M	85+	Blood	North West England
PHEGBS0122	ERR1741539	Ia	ST23	CC23	CC23	no	no	no	no	no	yes	no	*PI-1+PI-2a*	M	85+	Blood	South West England
200693	ERR3531627	III	**ST1219**	CC17	CC17	no	no	no	no	yes	no	yes	*PI-1+PI-2b*	M	65-84	Blood	South East England
141439	ERR3531632	Ib	ST10	CC8/CC10	CC8/CC10	no	no	yes	yes	no	yes	no	*PI-1 +PI-2a*	M	65-84	Blood	North East England
PHEGBS0393	ERR1741475	Ib	ST12	CC8/CC10	CC8/CC10	no	no	yes	yes	no	yes	no	*PI-1 +PI-2a*	F	65-84	Blood	Yorkshire and the Humber
PHEGBS0097	ERR1741888	Ib	ST104	CC8/CC10	singleton	no	no	yes	yes	no	yes	no	*PI-1 +PI-2a*	F	85+	Blood	Wales
PHEGBS0072	ERR1741514	Ib	ST15	CC8/CC10	singleton	no	no	yes	yes	no	yes	no	*PI-1 +PI-2a*	M	18-44	Blood	West Midlands
200706	ERR3531625	Ib	ST12	CC8/CC10	CC8/CC10	no	no	yes	yes	no	yes	no	*PI-1 +PI-2a*	F	45-64	Blood	Greater London
PHEGBS0670	ERR1742049	Ib	ST1	CC1	CC1	no	yes	no	no	no	yes	no	*PI-1+PI-2a*	F	45-64	Blood	Greater London
PHEGBS0145	ERR1741818	Ib	ST12	CC8/CC10	CC8/CC10	no	no	yes	yes	no	yes	no	*PI-1 +PI-2a*	M	65-84	Blood	Yorkshire and the Humber
PHEGBS0068	ERR1741595	Ib	ST12	CC8/CC10	CC8/CC10	no	no	yes	yes	no	yes	no	*PI-1 +PI-2a*	F	18-44	Blood	North West England
PHEGBS0667	ERR1741656	Ib	ST12	CC8/CC10	CC8/CC10	no	no	yes	yes	no	yes	no	*PI-1 +PI-2a*	M	65-84	Blood	South East England
PHEGBS0450	ERR1741493	Ib	ST1	CC1	CC1	no	yes	no	no	no	yes	no	*PI-1+PI-2a*	F	65-84	Blood	North West England
PHEGBS0061	ERR1741474	Ib	ST8	CC8/CC10	CC8/CC10	no	no	yes	yes	no	yes	no	*PI-1 +PI-2a*	F	45-64	Blood	South East England
PHEGBS0411	ERR1742019	Ib	ST8	CC8/CC10	CC8/CC10	no	no	yes	yes	no	yes	no	*PI-1 +PI-2a*	F	18-44	Blood	Greater London
PHEGBS0555	ERR1741852	Ib	ST8	CC8/CC10	CC8/CC10	no	no	yes	yes	no	yes	no	*PI-1 +PI-2a*	F	18-44	Blood	North West England
PHEGBS0382	ERR1741847	Ib	ST8	CC8/CC10	CC8/CC10	no	no	yes	yes	no	yes	no	*PI-1 +PI-2a*	M	65-84	Blood	Yorkshire and the Humber
200698	ERR3531634	Ib	ST8	CC8/CC10	CC8/CC10	no	no	yes	yes	no	yes	no	*PI-1 +PI-2a*	M	45-64	Blood	Wales
PHEGBS0135	ERR1741673	Ib	ST8	CC8/CC10	CC8/CC10	no	no	yes	yes	no	yes	no	*PI-1 +PI-2a*	M	65-84	Blood	South East England
PHEGBS0622	ERR1741630	Ib	ST8	CC8/CC10	CC8/CC10	no	no	yes	yes	no	yes	no	*PI-1 +PI-2a*	M	65-84	Blood	South East England
PHEGBS0428	ERR1741389	Ib	ST8	CC8/CC10	CC8/CC10	no	no	yes	yes	no	yes	no	*PI-1 +PI-2a*	M	85+	Blood	South East England
PHEGBS0568	ERR1741647	II	ST1	CC1	CC1	no	yes	no	no	no	yes	no	*PI-1+PI-2a*	F	45-64	Blood	South East England
200683	ERR3531633	Ib	ST8	CC8/CC10	CC8/CC10	no	no	yes	yes	no	yes	no	*PI-1 +PI-2a*	M	65-84	Blood	Wales
PHEGBS0398	ERR1741711	II	**ST1213**	CC22/CC1213	CC22/CC1213	no	no	no	no	no	yes	no	*PI-1+PI+2a*	M	85+	Blood	North West England
PHEGBS0080	ERR1741859	II	ST22	CC22/CC1213	CC22/CC1213	no	no	no	no	no	yes	no	*PI-1+PI+2a*	M	65-84	Blood	Scotland
PHEGBS0154	ERR1741575	Ib	ST9	CC8/CC10	CC8/CC10	no	no	yes	yes	no	yes	no	*PI-1 +PI-2a*	M	65-84	Blood	not available
PHEGBS0151	ERR1741944	II	ST10	CC8/CC10	CC8/CC10	no	no	yes	yes	no	yes	no	*PI-1 +PI-2a*	M	18-44	Blood	Yorkshire and the Humber
PHEGBS0308	ERR1741488	II	ST12	CC8/CC10	CC8/CC10	no	no	yes	yes	no	yes	no	*PI-1 +PI-2a*	F	65-84	Blood	Yorkshire and the Humber
PHEGBS0549	ERR1741850	II	ST1	CC1	CC1	no	yes	no	no	no	yes	no	*PI-1+PI-2a*	F	18-44	Blood	Greater London
PHEGBS0091	ERR1741407	II	ST12	CC8/CC10	CC8/CC10	no	no	yes	yes	no	yes	no	*PI-1 +PI-2a*	F	45-64	Blood	North West England
PHEGBS0041	ERR1742042	V	ST1	CC1	CC1	no	yes	no	no	no	yes	no	*PI-1+PI-2a*	M	65-84	Blood	Northern Ireland
PHEGBS0509	ERR1741644	II	ST12	CC8/CC10	CC8/CC10	no	no	yes	yes	no	yes	no	*PI-1 +PI-2a*	F	18-44	Blood	Greater London
PHEGBS0139	ERR1741954	V	ST19	CC19	CC19	no	no	no	no	no	yes	no	*PI-1+PI-2a*	M	45-64	Blood	South East England
PHEGBS0127	ERR1741367	V	ST1	CC1	CC1	no	yes	no	no	no	yes	no	*PI-1+PI-2a*	M	85+	Blood	Yorkshire and the Humber
PHEGBS0738	ERR1741902	V	ST19	CC19	CC19	no	no	no	no	no	yes	no	*PI-1+PI-2a*	M	45-64	Tissue	Greater London
PHEGBS0595	ERR1741483	V	ST19	CC19	CC19	no	no	no	no	no	yes	no	*PI-1+PI-2a*	M	65-84	Blood	Greater London
PHEGBS0608	ERR1741534	V	ST19	CC19	CC19	no	no	no	no	no	yes	no	*PI-1+PI-2a*	F	18-44	Blood	Greater London
PHEGBS0657	ERR1741517	V	ST19	CC19	CC19	no	no	no	no	no	yes	no	*PI-1+PI-2a*	F	18-44	Blood	Greater London
PHEGBS0132	ERR1741466	III	ST529	singleton	CC19	no	no	no	no	no	no	no	*PI-1+PI-2a*	F	45-64	Blood	Greater London
PHEGBS0171	ERR1741963	II	ST12	CC8/CC10	CC8/CC10	no	no	yes	yes	no	yes	no	*PI-1 +PI-2a*	F	18-44	Blood	South West England
PHEGBS0491	ERR1741535	II	ST12	CC8/CC10	CC8/CC10	no	no	yes	yes	no	yes	no	*PI-1 +PI-2a*	F	65-84	Blood	South East England
PHEGBS0639	ERR1741887	V	ST110	CC19	CC19	no	no	no	no	yes	yes	no	*PI-1+PI-2a*	M	45-64	Blood	Greater London
PHEGBS0092	ERR1741722	II	ST12	CC8/CC10	CC8/CC10	no	no	yes	yes	no	yes	no	*PI-1 +PI-2a*	M	65-84	Blood	Greater London
PHEGBS0390	ERR1741684	II	ST12	CC8/CC10	CC8/CC10	no	no	yes	yes	no	yes	no	*PI-1 +PI-2a*	M	18-44	Blood	South West England
PHEGBS0360	ERR1742032	V	ST1	CC1	CC1	no	yes	no	no	no	yes	no	*PI-1+PI-2a*	M	65-84	Blood	Greater London

Table S2 (continued).

Sample ID	ERA number	Serotype	ST	CC_MLST	CC_wgs#	*alp2*	*alp3*	*bca*	*cba*	*rib*	*bibA*	*hvgA*	Pilus island genes	Sex	Age group	Specimen	Region
PHEGBS0581	ERR1741660	II	ST12	CC8/CC10	CC8/CC10	no	no	yes	yes	no	yes	no	*PI-1 +PI-2a*	M	18-44	Blood	South West England
PHEGBS0153	ERR1741822	II	ST104	CC8/CC10	singleton	no	no	yes	yes	no	yes	no	*PI-1 +PI-2a*	F	65-84	Blood	North West England
PHEGBS0598	ERR1741680	III	**ST1316**	CC19	CC19	no	no	no	no	yes	yes	no	*PI-1+PI-2a*	F	65-84	Blood	Greater London
PHEGBS0253	ERR1741828	II	ST19	CC19	CC19	no	no	no	no	yes	yes	no	*PI-1+PI-2a*	F	18-44	Placenta	North West England
PHEGBS0586	ERR1741614	II	ST652	CC8/CC10	CC8/CC10	no	no	yes	yes	no	yes	no	*PI-1 +PI-2a*	M	18-44	pus	Yorkshire and the Humber
PHEGBS0394	ERR1741769	II	ST9	CC8/CC10	CC8/CC10	no	no	yes	yes	no	yes	no	*PI-1 +PI-2a*	F	45-64	Blood	South East England
PHEGBS0296	ERR1741462	III	ST19	CC19	CC19	no	no	no	no	yes	yes	no	*PI-1+PI-2a*	M	45-64	Blood	East England
PHEGBS0566	ERR1741860	III	ST19	CC19	CC19	no	no	no	no	yes	yes	no	*PI-1+PI-2a*	F	65-84	Blood	South East England
PHEGBS0575	ERR1741521	Ib	**ST1220**	CC8/CC10	CC17	no	no	yes	yes	no	yes	no	*PI-1 +PI-2a*	F	18-44	pus	Wales
PHEGBS0567	ERR1741438	III	ST19	CC19	CC19	no	no	no	no	yes	yes	no	*PI-1+PI-2a*	F	65-84	Abscess	South East England
PHEGBS0377	ERR1741496	III	ST19	CC19	CC19	no	no	no	no	yes	yes	no	*PI-1+PI-2a*	F	18-44	Blood	Scotland
PHEGBS0501	ERR1741911	III	**ST1221**	CC17	CC17	no	no	no	no	yes	no	yes	*PI-1+PI-2b*	F	18-44	Blood	Wales
PHEGBS0476	ERR1741458	IV	**ST1351**	CC17	CC17	no	no	no	no	yes	no	yes	*PI-1+PI-2b*	F	85+	Blood	South East England
PHEGBS0237	ERR1742117	III	ST17	CC17	CC17	no	no	no	no	yes	no	yes	*PI-1+PI-2b*	F	18-44	Placenta	South East England
PHEGBS0610	ERR1742130	III	ST17	CC17	CC17	no	no	no	no	yes	no	yes	*PI-1+PI-2b*	F	18-44	Blood	South East England
PHEGBS0359	ERR1741657	III	ST19	CC19	CC19	no	no	no	no	yes	yes	no	*PI-1+PI-2a*	F	85+	Blood	Yorkshire and the Humber
PHEGBS0286	ERR1741880	III	ST17	CC17	CC17	no	no	no	no	yes	no	yes	*PI-1+PI-2b*	F	18-44	Blood	Wales
PHEGBS0643	ERR1741746	III	ST19	CC19	CC19	no	no	no	no	yes	yes	no	*PI-1+PI-2a*	M	45-64	Blood	Greater London
PHEGBS0288	ERR1741473	III	ST17	CC17	CC17	no	no	no	no	yes	no	yes	*PI-1+PI-2b*	F	85+	Blood	Wales
PHEGBS0052	ERR1741861	III	ST17	CC17	CC17	no	no	no	no	yes	no	yes	*PI-1+PI-2b*	F	85+	Blood	South East England
PHEGBS0355	ERR1741387	III	ST17	CC17	CC17	no	no	no	no	yes	no	yes	*PI-1+PI-2b*	F	18-44	Blood	Yorkshire and the Humber
PHEGBS0626	ERR1741728	III	ST19	CC19	CC19	no	no	no	no	yes	yes	no	*PI-1+PI-2a*	F	18-44	Blood	Greater London
200710	ERR3589625	III	ST17	CC17	CC17	no	no	no	no	yes	no	yes	*PI-1+PI-2b*	F	18-44	Blood	Yorkshire and the Humber
PHEGBS0664	ERR1742011	III	ST19	CC19	CC19	no	no	no	no	yes	yes	no	*PI-1+PI-2a*	M	65-84	n/a	Greater London
PHEGBS0243	ERR1741633	III	ST17	CC17	CC17	no	no	no	no	yes	no	yes	*PI-1+PI-2b*	F	18-44	Blood	Yorkshire and the Humber
PHEGBS0372	ERR1741524	III	ST19	CC19	CC19	no	no	no	no	yes	yes	no	*PI-1+PI-2a*	F	18-44	Blood	East England
PHEGBS0480	ERR1741862	III	ST17	CC17	CC17	no	no	no	no	yes	no	yes	*PI-1+PI-2b*	F	18-44	Blood	Greater London
PHEGBS0144	ERR1741588	III	ST17	CC17	CC17	no	no	no	no	yes	no	yes	*PI-1+PI-2b*	F	18-44	Blood	Greater London
PHEGBS0492	ERR1741922	III	ST19	CC19	CC19	no	no	no	no	yes	yes	no	*PI-1+PI-2a*	F	85+	Blood	East England
PHEGBS0524	ERR1741696	III	ST23	CC23	CC23	yes	no	no	no	no	yes	no	*PI-1+PI-2a*	M	65-84	Blood	Greater London
PHEGBS0275	ERR1742034	III	ST17	CC17	CC17	no	no	no	no	yes	no	yes	*PI-1+PI-2b*	F	18-44	Blood	Greater London
PHEGBS0483	ERR1741662	III	**ST1212**	singleton	singleton	no	no	no	no	no	yes	no	*PI-1+PI-2b*	F	18-44	Blood	Greater London
PHEGBS0561	ERR1741542	III	ST17	CC17	CC17	no	no	no	no	yes	no	yes	*PI-1+PI-2b*	F	18-44	Blood	Greater London
PHEGBS0559	ERR1741492	III	ST17	CC17	CC17	no	no	no	no	yes	no	yes	*PI-1+PI-2b*	F	18-44	Blood	Greater London
PHEGBS0252	ERR1741658	III	ST19	CC19	CC19	no	no	no	no	yes	yes	no	*PI-1+PI-2a*	F	18-44	Blood	South East England
PHEGBS0416	ERR1741402	III	ST17	CC17	CC17	no	no	no	no	yes	no	yes	*PI-1+PI-2b*	F	18-44	Blood	Greater London
PHEGBS0156	ERR1741857	III	ST19	CC19	CC19	no	no	no	no	yes	yes	no	*PI-1+PI-2a*	F	45-64	Blood	West Midlands
PHEGBS0071	ERR1742139	III	ST19	CC19	CC19	no	no	no	no	yes	yes	no	*PI-1+PI-2a*	M	45-64	Blood	North East England
200697	ERR3531629	III	ST17	CC17	CC17	no	no	no	no	yes	no	yes	*PI-1+PI-2b*	F	18-44	Blood	Greater London
PHEGBS0176	ERR1742030	III	ST23	CC23	CC23	yes	no	no	no	no	yes	no	*PI-1+PI-2a*	F	18-44	Blood	South West England
PHEGBS0577	ERR1741835	III	ST17	CC17	CC17	no	no	no	no	yes	no	yes	*PI-1+PI-2b*	F	85+	Blood	Greater London
PHEGBS0207	ERR1741997	III	ST17	CC17	CC17	no	no	no	no	yes	no	yes	*PI-1+PI-2b*	F	85+	Blood	South West England
200694	ERR3531635	III	ST17	CC17	CC17	no	no	no	no	yes	no	yes	*PI-1+PI-2b*	M	65-84	Blood	South East England
PHEGBS0513	ERR1741701	III	ST17	CC17	CC17	no	no	no	no	yes	no	yes	*PI-1+PI-2b*	F	18-44	Blood	South West England
PHEGBS0389	ERR1741939	III	ST17	CC17	CC17	no	no	no	no	yes	no	yes	*PI-1+PI-2b*	F	18-44	Blood	South East England
PHEGBS0117	ERR1741635	III	ST17	CC17	CC17	no	no	no	no	yes	no	yes	*PI-1+PI-2b*	F	18-44	Blood	South East England
PHEGBS0380	ERR1741867	III	ST17	CC17	CC17	no	no	no	no	yes	no	yes	*PI-1+PI-2b*	F	18-44	Blood	South East England
PHEGBS0593	ERR1741754	III	ST19*	CC19	CC19	no	no	no	no	yes	yes	no	*PI-1+PI-2a*	M	18-44	Blood	North East England

Table S2 (continued).

Sample ID	ERA number	Serotype	ST	CC_MLST	CC_wgs#	*alp2*	*alp3*	*bca*	*cba*	*rib*	*bibA*	*hvgA*	Pilus island genes	Sex	Age group	Specimen	Region
PHEGBS0066	ERR1741366	III	ST17	CC17	CC17	no	no	no	no	yes	no	yes	*PI-1+PI-2b*	F	65-84	Blood	South East England
PHEGBS0429	ERR1741515	III	ST17	CC17	CC17	no	no	no	no	yes	no	yes	*PI-1+PI-2b*	F	85+	Blood	South East England
200682	ERR3531626	III	ST27	CC19	CC19	no	no	no	no	yes	yes	no	*PI-1+PI-2a*	F	18-44	Blood	South East England
PHEGBS0318	ERR1741792	III	ST17	CC17	CC17	no	no	no	no	yes	no	yes	*PI-1+PI-2b*	F	85+	Blood	South West England
PHEGBS0383	ERR1741449	III	ST17	CC17	CC17	no	no	no	no	yes	no	yes	*PI-1+PI-2b*	F	18-44	Blood	South East England
PHEGBS0408	ERR1741526	II	ST28	CC19	CC19	no	no	no	no	yes	yes	no	*PI-1+PI-2a*	F	18-44	Blood	Greater London
200696	ERR3531628	III	ST550	CC17	CC17	no	no	no	no	yes	no	yes	*PI-1+PI-2b*	F	18-44	Blood	Yorkshire and the Humber
PHEGBS0054	ERR1742070	III	ST283	singleton	singleton	no	no	yes	no	no	no	no	*PI-1+PI+2a*	M	65-84	Blood	North West England
PHEGBS0464	ERR1741702	II	ST28	CC19	CC19	no	no	no	no	yes	yes	no	*PI-1+PI-2a*	F	18-44	Blood	Greater London
PHEGBS0624	ERR1741580	II	ST28	CC19	CC19	no	no	no	no	yes	yes	no	*PI-1+PI-2a*	F	18-44	Blood	Greater London
PHEGBS0128	ERR1742140	V	ST1	CC1	CC1	no	yes	no	no	no	yes	no	*PI-1+PI-2a*	M	65-84	Blood	Greater London
PHEGBS0599	ERR1741659	V	ST1	CC1	CC1	no	yes	no	no	no	yes	no	*PI-1+PI-2a*	M	85+	Blood	Greater London
PHEGBS0373	ERR1741478	V	ST1	CC1	CC1	no	yes	no	no	no	yes	no	*PI-1+PI-2a*	M	65-84	Blood	Wales
PHEGBS0520	ERR1741361	V	ST1	CC1	CC1	no	yes	no	no	no	yes	no	*PI-1+PI-2a*	F	65-84	Blood	South East England
PHEGBS0219	ERR1741590	III	ST550	CC17	CC17	no	no	yes	no	yes	no	yes	*PI-1+PI-2b*	F	85+	Blood	South West England
PHEGBS0081	ERR1741497	V	ST1	CC1	CC1	no	yes	no	no	no	yes	no	*PI-1+PI-2a*	F	85+	Blood	Yorkshire and the Humber
PHEGBS0552	ERR1741992	IX	**ST1216**	CC130/CC1216	CC130/CC1216	no	no	yes	yes	no	yes	no	*PI-1 +PI-2a*	M	85+	Blood	Greater London
PHEGBS0042	ERR1742087	IX	ST130	CC130/CC1216	CC130/CC1216	no	no	yes	yes	no	yes	no	*PI-1 +PI-2a*	F	65-84	Blood	Northern Ireland
PHEGBS0592	ERR1741448	IX	ST130	CC130/CC1216	CC130/CC1216	no	no	yes	yes	no	yes	no	*PI-1 +PI-2a*	F	65-84	Blood	Greater London
PHEGBS0630	ERR1741525	V	ST498	CC498/CC24	CC23	no	no	yes	no	no	yes	no	*PI-1+PI-2a*	M	65-84	Blood	South East England
PHEGBS0399	ERR1741537	II	ST28	CC19	CC19	no	no	no	no	yes	yes	no	*PI-1+PI-2a*	F	65-84	Blood	Greater London
PHEGBS0337	ERR1741620	V	ST498	CC498/CC24	CC23	no	no	yes	no	no	yes	no	*PI-1+PI-2a*	M	85+	Blood	North West England
PHEGBS0648	ERR1741752	V	ST1	CC1	CC1	no	yes	no	no	no	yes	no	*PI-1+PI-2a*	F	18-44	Blood	Yorkshire and the Humber
PHEGBS0467	ERR1741981	V	ST1	CC1	CC1	no	yes	no	no	yes	yes	no	*PI-1+PI-2a*	F	18-44	Blood	Yorkshire and the Humber
PHEGBS0306	ERR1741485	V	ST1	CC1	CC1	no	yes	no	no	no	yes	no	*PI-1+PI-2a*	F	18-44	Blood	Greater London
PHEGBS0554	ERR1741388	II	ST28	CC19	CC19	no	no	no	no	yes	yes	no	*PI-1+PI-2a*	F	18-44	Blood	North West England
PHEGBS0532	ERR1741494	II	ST28	CC19	CC19	no	no	no	no	yes	yes	no	*PI-1+PI-2a*	F	18-44	Blood	South West England
PHEGBS0551	ERR1741634	II	ST28	CC19	CC19	no	no	no	no	yes	yes	no	*PI-1+PI-2a*	M	65-84	Blood	South West England
PHEGBS0378	ERR1741669	V	ST1	CC1	CC1	no	yes	no	no	no	yes	no	*PI-1+PI-2a*	F	18-44	Blood	Greater London
PHEGBS0164	ERR1741749	V	ST1	CC1	CC1	no	yes	no	no	no	yes	no	*PI-1+PI-2a*	M	45-64	Blood	South West England
PHEGBS0098	ERR1741573	V	ST1	CC1	CC1	no	yes	no	no	no	yes	no	*PI-1+PI-2a*	M	18-44	Blood	South West England
PHEGBS0368	ERR1742016	V	ST1	CC1	CC1	no	yes	no	no	no	yes	no	*PI-1+PI-2a*	F	18-44	Blood	Greater London
200690	ERR3531636	V	ST1	CC1	CC1	no	yes	no	no	no	yes	no	*PI-1+PI-2a*	F	85+	Blood	South West England
PHEGBS0082	ERR1741501	V	ST1	CC1	CC1	no	yes	no	no	no	yes	no	*PI-1+PI-2a*	F	85+	Blood	South West England
PHEGBS0446	ERR1741823	VI	ST1	CC1	CC1	no	no	yes	no	no	no	no	*PI-1+PI-2a*	M	65-84	Blood	South East England
PHEGBS0662	ERR1741427	VI	ST1	CC1	CC1	no	no	yes	no	no	no	no	*PI-1+PI-2a*	F	45-64	Blood	North East England
PHEGBS0556	ERR1741948	II	ST28	CC19	CC19	no	no	no	no	yes	yes	no	*PI-1+PI-2a*	M	65-84	Blood	South West England
PHEGBS0635	ERR1741616	V	ST26	CC26	CC26	no	no	no	no	yes	yes	no	*PI-1+PI+2a*	M	45-64	Throat swab	South East England
200684	ERR3531637	Ib	**ST1215**	singleton	CC1	no	yes	no	no	no	no	no	*PI-1+PI-2a*	M	65-84	Blood	South West England
PHEGBS0493	ERR1741913	V	**ST1217**	CC1	CC1	no	yes	no	no	no	yes	no	*PI-1+PI-2a*	F	18-44	Blood	South West England
PHEGBS0090	ERR1742102	V	**ST1314**	CC1	CC1	no	yes	no	no	no	yes	no	*PI-1+PI-2a*	M	45-64	CSF	North West England
100414	ERR2560245	V	**ST1350**	CC1	CC1	no	yes	no	no	no	yes	no	*PI-1+PI-2a*	F	18-44	Blood	South West England
200699	ERR3531631	III	ST861	CC19	CC19	no	no	no	no	yes	yes	no	*PI-1+PI-2a*	F	18-44	Vaginal swab	Yorkshire and the Humber
PHEGBS0266	ERR1741584	IV	ST136	CC1	CC1	no	no	no	no	no	yes	no	*PI-1+PI-2a*	F	18-44	Blood	Greater London
PHEGBS0533	ERR1741445	VI	ST14	CC1	CC1	no	no	no	no	no	yes	no	*PI-1+PI-2b*	F	18-44	Placenta	Wales
PHEGBS0463	ERR1742031	IV	ST196	CC1	CC1	no	no	no	no	no	yes	no	*PI-1+PI-2a*	F	18-44	Blood	Greater London
PHEGBS0448	ERR1741564	IV	ST196	CC1	CC1	no	no	no	no	no	yes	no	*PI-1+PI-2a*	F	65-84	Blood	South West England
PHEGBS0206	ERR1741687	IV	ST196	CC1	CC1	no	no	no	no	no	yes	no	*PI-1+PI-2a*	M	65-84	Blood	South West England
PHEGBS0627	ERR1741512	II	ST2	CC1	CC1	no	no	no	no	no	yes	no	*PI-1+PI-2a*	F	65-84	Blood	Yorkshire and the Humber
PHEGBS0100	ERR1741744	II	ST3	singleton	CC1	no	no	yes	no	no	no	no	*PI-1+PI-2a*	F	45-64	Blood	North East England
PHEGBS0084	ERR1741695	IV	ST459	CC1	CC1	no	no	no	no	no	yes	no	*PI-1+PI-2a*	F	85+	Blood	Greater London

Supplementary Figures

Figure S1. Phylogenetic relationship between UK ST1 isolates and previously reported recombined Portugal and Canadian ST1 non-serotype V isolates. Maximum likelihood phylogenetic tree of UK (n=23), Portugal (n=1, [1]) and Canada (n=2, [2]) GBS isolates based on 1,964 core SNPs with reference sequence SS1 (NZ_CP010867.1). Majority of UK ST1 isolates were serotype V (n=17), followed by serotype Ib, II and VI (n=2 per serotype), Portugal ST1 isolate (SH5446) was reported as serotype Ib, Canadian isolates (NGBS748) and (NGBS209) were serotypes II and VI, respectively. From left to right: mid-point rooted maximum likelihood phylogeny tree (with recombination regions removed), followed by information on serotype, and country of isolate origin. Tree scale indicates distance in SNPs.

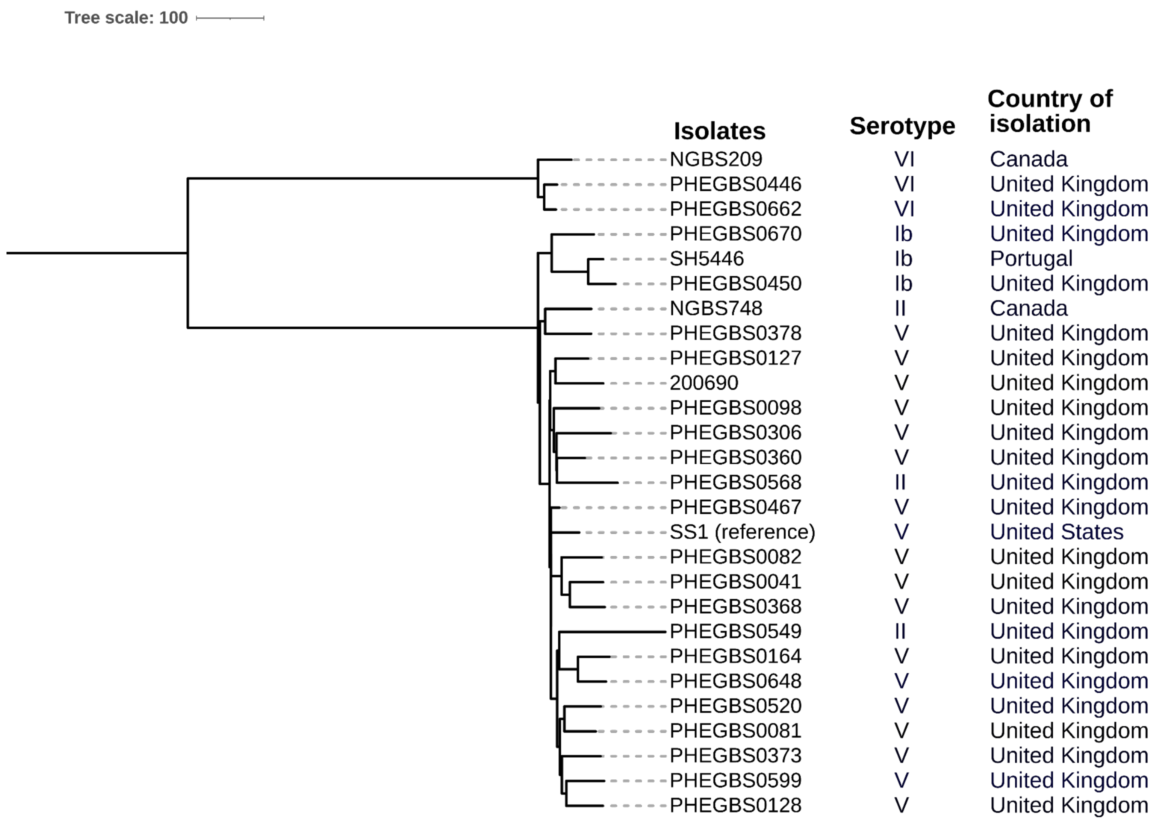

Figure S2. Comparison of recombination events among selected GBS groups. BRIG [3] was used to show Single Nucleotide Polymorphisms (SNPs) distribution **(a) among ST1 serotypes Ib, II and VI.** ST1 isolates of serotype Ib (n=3), II (n=3) and VI (n=3) from UK, Portugal and Canada were compared against reference strain SS1 (NZ_CP010867.1). From inner to outermost circle: inner circle (black line) is reference SS1, then polymorphisms identified in each of the GBS isolates: serotype Ib (red – PHEGBS0670, UK; aqua - PHEGBS0450, UK; and magenta – SH5446, Portugal [1]); serotype II (olive - PHEGBS0549, UK; purple - PHEGBS0568, UK; and teal – NGBS748, Canada [2]); serotype VI (blue – PHEGBS662, UK; green – PHEGBS446, UK; and orange – NGBS209, Canada. **(b) among ST19 serotype V isolates.** ST19 serotype V isolates (n=5) identified in the study were compared to the ST19 serotype III reference genome SG-M25 (NZ_CP021867.1). From inner to outermost circle: inner circle (black line) is reference SG-M25, then polymorphisms identified in each of the GBS isolates. Outermost circle indicates genome landmarks as follows: cps locus - capsular polysaccharide locus (in orange), *alp*3 - alpha like surface protein encoding gene (in green), seven multi-locus housekeeping genes (*adhP*, *atr*, *tkt*, *glcK*, *sdhA*, *glnA*, and *pheS*, in purple), MGE - Mobile genetic elements and pilus island genes *PI-1* and *PI-2a* (in black).

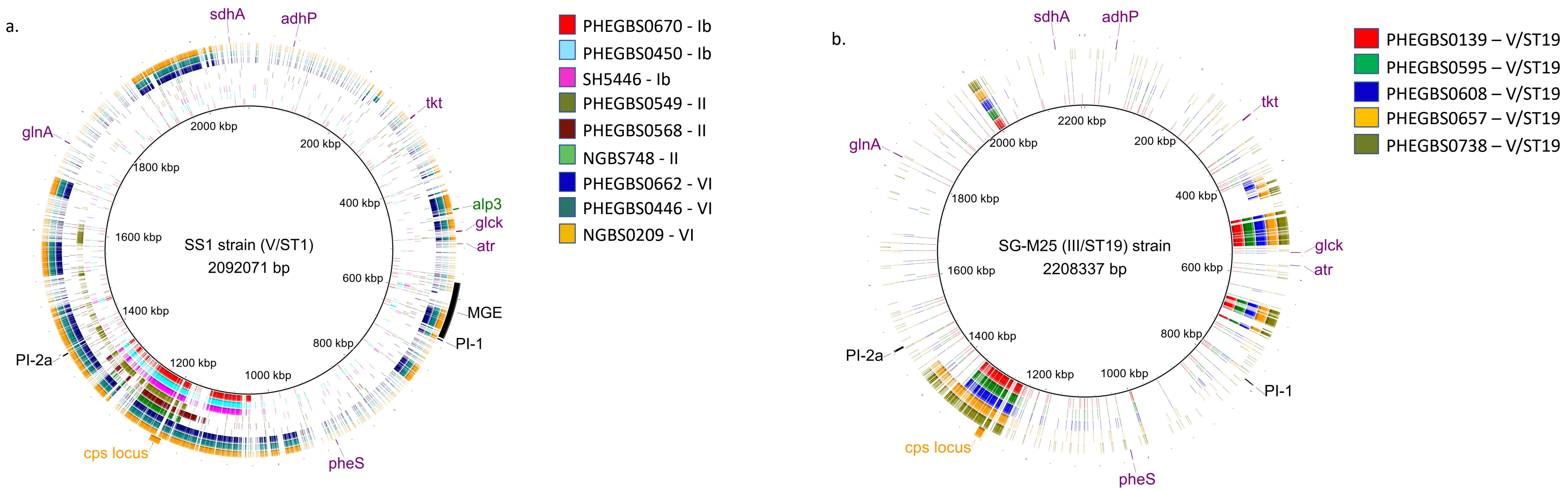
a.
sdhA
adhP
tkt
glnA
alp3
glck
atr
MGE
PI-1
PI-2a
cps locus
pheS
2000 kbp
200 kbp
1800 kbp
400 kbp
1600 kbp
600 kbp
1400 kbp
800 kbp
1200 kbp
1000 kbp
SS1 strain (V/ST1)
2092071 bp
PHEGBS0670 - Ib
PHEGBS0450 - Ib
SH5446 - Ib
PHEGBS0549 - II
PHEGBS0568 - II
NGBS748 - II
PHEGBS0662 - VI
PHEGBS0446 - VI
NGBS0209 - VI
b.
sdhA
adhP
tkt
glnA
glck
atr
PI-2a
PI-1
cps locus
pheS
2200 kbp
2000 kbp
200 kbp
1800 kbp
400 kbp
1600 kbp
600 kbp
1400 kbp
800 kbp
1200 kbp
1000 kbp
SG-M25 (III/ST19) strain
2208337 bp
PHEGBS0139 – V/ST19
PHEGBS0595 – V/ST19
PHEGBS0608 – V/ST19
PHEGBS0657 – V/ST19
PHEGBS0738 – V/ST19

58

Figure S3: SNPs distribution in conserved *cpsE-cpsG* and *neuB-neuA* regions of *cpsIV* locus of CC1 and CC17 GBS isolates from Ireland, France and England and Wales. Mapping, SNP calling, and visualization was performed by Geneious 11.0.5 using entire *cpsIV* sequences of current study CC1 and CC17 serotype IV isolates (PHEGBS0084, PHEGBS0206, PHEGBS0463, PHEGBS0448, PHEGBS0266 and PHEGBS0476) and available partially (*cpsE*, *cpsG*, *neuB* and *neuA*) and fully sequenced (*cpsF*, *cpsG*, *neuC* and *neuD*) sequences of Irish CC1 and CC17 serotype IV isolates (GBS148, GBS150 and GBS175) [4] using a reference full cpsIV locus of French CC17 serotype IV isolate (CCH209361)[5]. Different colours are used to highlight important landmarks in *cpsIV* locus of CC209361 with yellow representing *cps*(A-D, H-M) genes, red showing conserved *cps*(E,F,G) and *neu*(B, C, D and A) genes respectively. Grey region showing similarity whereas each vertical black line representing a single-nucleotide polymorphism (SNP) in each isolate against a reference *cpsIV* locus.

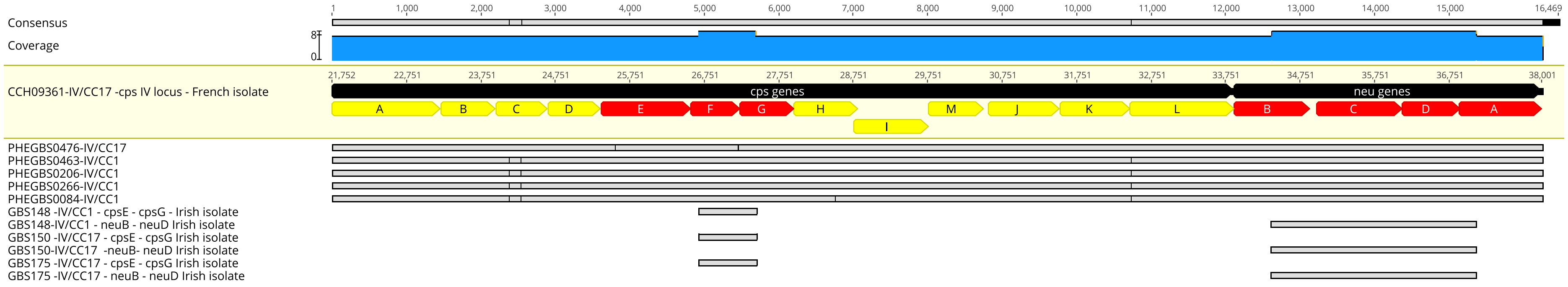

References

[1] Lopes E, Fernandes T, Machado MP, Carriço JA, Melo-Cristino J, Ramirez M, et al. Increasing macrolide resistance among Streptococcus agalactiae causing invasive disease in non-pregnant adults was driven by a single capsular-transformed lineage, Portugal, 2009 to 2015. Eurosurveillance 2018;23. https://doi.org/10.2807/1560-7917.ES.2018.23.21.1700473.

[2] Neemuchwala A, Teatero S, Athey TBT, McGeer A, Fittipaldi N. Capsular switching and other large-scale recombination events in invasive sequence type 1 group B Streptococcus. Emerg Infect Dis 2016;22:1941–4. https://doi.org/10.3201//eid2211.152064.

[3] Alikhan NF, Petty NK, Ben Zakour NL, Beatson SA. BLAST Ring Image Generator (BRIG): Simple prokaryote genome comparisons. BMC Genomics 2011;12. https://doi.org/10.1186/1471-2164-12-402.

[4] Teatero S, McGeer A, Low DE, Li A, Demczuk W, Martin I, et al. Characterization of invasive group B Streptococcus strains from the greater Toronto area, Canada. J Clin Microbiol 2014;52:1441–7. https://doi.org/10.1128/JCM.03554-13.

[5] Bellais S, Six A, Fouet A, Longo M, Dmytruk N, Glaser P, et al. Capsular switching in group B streptococcus CC17 hypervirulent clone: A future challenge for polysaccharide vaccine development. J Infect Dis 2012;206:1745–52. https://doi.org/10.1093/infdis/jis605.
